# Sex-specific moderation by lifestyle and psychosocial factors on the genetic contributions to adiposity in 112,151 individuals from UK Biobank

**DOI:** 10.1038/s41598-018-36629-0

**Published:** 2019-01-23

**Authors:** Catherine M. Calvin, Saskia P. Hagenaars, John Gallacher, Sarah E. Harris, Gail Davies, David C. Liewald, Catharine R. Gale, Ian J. Deary

**Affiliations:** 1 Dementias Platform UK, Department of Psychiatry, University of Oxford Warneford Hospital, Oxford, UK; 2 Centre for Cognitive Ageing and Cognitive Epidemiology (CCACE), Department of Psychology, University of Edinburgh, Edinburgh, UK; 3 Social Genetic and Developmental Psychiatry Research Centre, Institute of Psychiatry, Psychology & Neuroscience, King’s College London, Denmark Hill, London, UK; 4 Department of Psychology, University of Edinburgh, 7 George Square, Edinburgh, UK; 5 Medical Genetics Section, University of Edinburgh Centre for Genomic and Experimental Medicine and MRC Institute of Genetics and Molecular Medicine, Western General Hospital, Crewe Road, Edinburgh, UK; 6 MRC Lifecourse Epidemiology Unit, University of Southampton, Southampton, UK

## Abstract

Evidence suggests that lifestyle factors, e.g. physical activity, moderate the manifestation of genetic susceptibility to obesity. The present study uses UK Biobank data to investigate interaction between polygenic scores (PGS) for two obesity indicators, and lifestyle and psychosocial factors in the prediction of the two indicators, with attention to sex-specific effects. Analyses were of 112 151 participants (58 914 females; 40 to 73 years) whose genetic data passed quality control. Moderation effects were analysed in linear regression models predicting body mass index (BMI) and waist-to-hip ratio (WHR), including interaction terms for PGS and each exposure. Greater physical activity, more education, higher income, moderate *vs* low alcohol consumption, and low material deprivation were each associated with a relatively lower risk for manifestation of genetic susceptibility to obesity (p < 0.001); the moderating effects of physical activity and alcohol consumption were greater in women than men (three-way interaction: p = 0.009 and p = 0.008, respectively). More income and less neuroticism were related to reduced manifestation of genetic susceptibility to high WHR (p = 0.007; p = 0.003); the effect of income was greater in women (three-way interaction: p = 0.001). Lifestyle and psychosocial factors appear to offset genetic risk for adiposity in mid to late adulthood, with some sex-specific associations.

## Introduction

Overweight and obesity are major risk factors for non-communicable disease (e.g. coronary heart disease, stroke, type 2 diabetes, specific cancers, dementia), metabolic disturbance, psychological disorder, and premature death^1^. The rising global prevalence of obesity, largely attributable to changes in lifestyle factors, comes at huge personal and economic cost to existing and future generations^2^. In Britain alone, where rates are the highest in Europe^2^, obesity is currently responsible for one in ten deaths and has direct costs to the NHS of upwards of £5 billion a year^3^; indirect costs to employers and household incomes are reported to be double that figure^4^. Whereas a key challenge for societies is to target lifestyle factors and aspects of an obesogenic environment that contribute to obesity risk, there is a genetic contribution to obesity. Between 40 to 60% of the variation in body mass index (BMI), and 30–60% of the variation in waist-to-hip ratio (WHR) is attributable to genetic effects^5,6^. Advances in candidate gene and genome-wide association studies (GWAS) have so far identified dozens of susceptibility gene loci associated with BMI^7,8^. Validated loci (e.g., *FTO*) explain only a small proportion of known heritability, together accounting for 1%-2% of variance in BMI^5,8^. However, using information from all available (n = 586,898) autosomal single nucleotide polymorphisms (SNPs) has greatly increased the proportion of variance in BMI (17%) explained^9^.

Emerging big-data cohorts are providing new opportunities to concurrently test multiple genetic and environmental factors in risk models of obesity, as well as consider their interaction. So far, replicated studies report evidence that greater physical activity lowers the manifestation of genetic susceptibility to higher BMI^10–19^; for example, a recent meta-analysis estimated that the contribution of the *FTO* risk allele to the odds of obesity risk could be attenuated by 27% in physically active versus sedentary adults^11^. Additional protective lifestyle factors such as low-salt diet^10^ and non-smoking^18,19^ are also reported to attenuate genetic influences on BMI, as well as increased alcohol consumption^20^ or frequency^10,19^. More recently, evidence has been reported from ~120,000 men and women recruited into UK Biobank that high versus low material deprivation inflated the association between a genetic risk score for obesity (a composite of 69 risk variants) and measured BMI (the interaction effect was *p* = 2 × 10^−10^)^8^, thereby extending the range of factors in the obesogenic environment that might moderate genetic influences on adult adiposity. Indeed, in a later ‘hypothesis-free’ investigation of the same cohort, among 15 of 131 environmental exposures that showed significant statistical interaction with genetic risk for BMI (composite of 94 risk variants), were items measuring psychosocial factors, such as self-reported depression and ‘fed-up feelings’, as well as socioeconomic factors (income and deprivation) and health-related lifestyle behaviours (physical activity, smoking, alcohol)^19^.

Due to the sex-specific characteristics of anthropometric indicators for obesity and some of their putative moderators (e.g. smoking, alcohol), and reported sex interaction effects in the associations between specific SNPs and body fat distribution^21,22^, in the present study we have undertaken analyses separately for men and women of a range of potential lifestyle and psychosocial moderators on genetic susceptibility to adiposity. Whereas the relevant literature consistently reports on analyses that account for sex differences, in the present study we draw upon UK Biobank data that are sufficiently large to enable separate analyses in men and women. The data on this cohort also allow us to consider a range of lifestyle and psychosocial variables, which show replicated associations with long-term health outcomes. These include cognitive ability and the personality trait neuroticism, both of which have been associated with mortality and morbidity risk, including CVD^23–26^, metabolic syndrome^27–29^, and obesity^29,30^, and which warrant investigation alongside socioeconomic risk factors for disease^31^. Childhood cognitive ability and personality traits were previously shown to moderate the manifestation of genetic risk for type 2 diabetes in a prospective cohort study of older adults living in Scotland^32,33^.

A second aim of the study is to report on moderators of the genetic susceptibility to abdominal fat accumulation (central obesity) as indicated by polygenic scores (PGS) for WHR, as well as overall obesity indexed by BMI. Evidence points to midlife WHR as a greater risk factor, relative to BMI and waist circumference, for certain morbidities, e.g. dementia^34^, and mortality in older adulthood^35,36^. This has relevance to UK Biobank’s participants whose baseline data for adiposity were collected at 40 to 73 years of age. Given that BMI in older adults may sometimes be protective to health in the context of increased risk of frailty and falls among the elderly^37^, and that BMI fails to discriminate between fat versus muscle weight, we include WHR in the present study as an enhancement to the study design. With evidence that the genetic loci associated with WHR are distinct from those associated with BMI^21^, we generate polygenic scores for BMI and WHR (with and without adjustment for BMI) separately to assess moderator effects on their respective associations with risk of overall and central adiposity.

## Results

### Moderating factors in relation to adult BMI and WHR in men and women

We first considered associations between lifestyle and psychosocial factors and the adiposity outcomes. Adult BMI and WHR were associated with each of the variables in men and women respectively (p ≤ 0.0018; see Table 1) with the exception being BMI and neuroticism among men. Male smokers were on average lower on BMI and higher on WHR than men who had never smoked, whereas men who had quit smoking were highest on both BMI and WHR relative to current and non-smokers. Female smokers were highest in mean WHR relative to past or non-smokers, and those who had quit were higher on WHR than those who had never smoked. Furthermore, women who had previously smoked were highest in BMI relative to current smokers and the never smoked group, but there was no difference in mean BMI between these two latter groups. Alcohol dose showed a curvilinear association with BMI and WHR in both sexes, with a point of inflection at ~10 grams/day; values below this were inversely associated with BMI, whereas values above were positively associated (Table 1; Fig. S1a). Therefore, participants who reported drinking 1–2 units per day showed the lowest BMI and WHR levels in men and women. This dosage category was used as the reference group in all subsequent regression models, and in models where alcohol was a continuous variable we excluded abstainers or low-level drinkers from the analyses to meet the assumption of linearity. The inverse linear association between physical activity and BMI and WHR, in men and women respectively, saw a threshold effect at ~1000–2000 MET mins/week, beyond which the gradient levelled off (Fig. S1b).

**Table 1:** Lifestyle and psychosocial factors in men and women: descriptive data and associations with adult BMI and WHR.

	Men				Women			
	N	Mean (SD)	BMI mean (SD)	WHR mean (SD)	N	Mean (SD)	BMI mean (SD)	WHR mean (SD)
Total Sample	53 235		27.94 (4.30)	0.938 (0.065)	58 911		27.15 (5.19)	0.819 (0.070)
*Smoking*								
never	26 174		27.55 (4.20)	0.927 (0.064)	33 723		26.88 (5.12)	0.810 (0.069)
previous	19 886		28.67 (4.25)	0.950 (0.064)	18 431		27.76 (5.24)	0.828 (0.070)
current	7041		27.27 (4.49)	0.943 (0.068)	6597		26.81 (5.25)	0.834 (0.071)
*Alcohol*								
grams/day		24.34 (0.08)				13.63 (0.06)		
Non-drinker	2727		28.38 (5.19)	0.945 (0.071)	4845		28.26 (6.12)	0.830 (0.077)
<8 g/day	6812		27.57 (4.39)	0.932 (0.069)	15 416		27.09 (5.14)	0.816 (0.070)
8–16 g/day	9122		27.49 (4.11)	0.930 (0.064)	13 472		26.42 (4.61)	0.810 (0.067)
16–24 g/day	7924		27.65 (3.97)	0.932 (0.062)	6801		26.40 (4.55)	0.815 (0.067)
>24 g/day	17 900		28.11 (4.04)	0.941 (0.062)	6289		26.70 (4.61)	0.824 (0.067)
*Physical activity*								
MET		3315 (4445)				2835 (3400)		
low	8020		28.88 (4.87)	0.954 (0.066)	7940		28.48 (5.88)	0.829 (0.072)
moderate	20 722		27.73 (4.13)	0.936 (0.064)	22 391		26.79 (4.89)	0.815 (0.069)
high	14 923		27.43 (3.88)	0.926 (0.063)	13 577		26.20 (4.58)	0.810 (0.068)
*Education*								
graduate	16 734		27.08 (3.96)	0.923 (0.063)	17 118		26.18 (4.85)	0.806 (0.068)
non-graduate	17 118		28.33 (4.39)	0.945 (0.065)	41 268		27.55 (5.27)	0.824 (0.070)
*Income*								
<£18 k	9826		28.29 (4.82)	0.955 (0.068)	12 319		27.98 (5.86)	0.834 (0.072)
£18-£31 k	11 969		27.95 (4.33)	0.942 (0.065)	13 092		27.38 (5.20)	0.822 (0.069)
£31-£52 k	13 025		27.84 (4.11)	0.933 (0.063)	12 588		26.93 (5.05)	0.812 (0.068)
£52-£100 k	10 346		27.66 (3.91)	0.926 (0.061)	8901		26.29 (4.77)	0.802 (0.066)
>£100 k	2658		27.41 (3.71)	0.916 (0.060)	2176		25.34 (4.37)	0.793 (0.064)
*Deprivation*								
score		−1.46 (3.0)				−1.52 (2.93)		
1^st^ quartile	13 406		27.64 (3.89)	0.932 (0.062)	14 596		26.57 (4.71)	0.811 (0.067)
2^nd^ quartile	13 211		27.86 (4.10)	0.935 (0.063)	14 791		26.85 (4.87)	0.814 (0.069)
3^rd^ quartile	13 077		27.97 (4.24)	0.938 (0.065)	14 923		27.14 (5.14)	0.818 (0.070)
4^th^ quartile	13 473		28.28 (4.87)	0.947 (0.069)	14 528		28.06 (5.85)	0.831 (0.073)
*Cognitive ability*								
score, 0–13		6.26 (2.17)				6.07 (2.04)		
high score	7852		27.67 (4.20)	0.934 (0.064)	7823		26.83 (5.11)	0.816 (0.070)
low score	9412		28.08 (4.33)	0.944 (0.066)	10 948		27.26 (5.25)	0.824 (0.072)
*Neuroticism*								
score, 0–12		3.53 (3.12)				4.45 (3.18)		
1^st^ quartile	17 147		27.91 (4.22)	0.935 (0.064)	18 465		27.04 (5.01)	0.816 (0.069)
2^nd^ quartile	11 811		27.90 (4.17)	0.938 (0.064)	12 636		27.02 (5.07)	0.817 (0.069)
3^rd^ quartile	12 499		28.03 (4.37)	0.941 (0.065)	15 153		27.16 (5.26)	0.818 (0.070)
4^th^ quartile	9505		27.98 (4.50)	0.942 (0.067)	10 817		27.51 (5.53)	0.823 (0.072)

Sex differences on each exposure were assessed using t-tests for continuous measures of exposures, or ANOVA or Pearson’s chi-square for categorical-only variables. Within sex groups, associations were tested between the adiposity indicators and each respective lifestyle or psychosocial factor using ANOVA (smoking, alcohol, physical activity, income, deprivation, neuroticism) or t-tests (education, cognitive ability). Significance p-values from 39 of 40 tests (8 × sex differences; 32 × adiposity differences) survived correction for FDR (p ≤ 0.0018); the exception was BMI differences across neuroticism quartiles (*p* = 0.0652).

Advantageous socioeconomic position was associated with lower average adiposity levels in men and women respectively (see Table 1). Firstly, college graduates were substantively lower on BMI and WHR than their non-graduate peers. Secondly, income showed a clear incremental inverse association with BMI and WHR respectively, in that higher income categories were associated with lower mean values on these adiposity measures, in men and women. Third, Townsend deprivation index showed a positive linear association with adiposity measures. There was an attenuation of the regression curve for BMI in men above average levels of deprivation (see Fig. S1c), and so subsequent moderation models included Townsend deprivation as quartile groupings.

Among psychological traits, cognitive ability showed an inverse pattern of association with adult BMI and WHR, in men and women (Fig. S1d). There was an attenuated gradient beyond the mean (score of ~6) for the association with male and female WHR, and with female BMI, but conversely, in men the weaker gradient for the association between cognitive ability and BMI was evident at low values, and beyond the mean the association became stronger. Neuroticism was positively associated with WHR in men and women and positively associated with BMI in women but not men (Fig. S1e).

### BMI and WHR polygenic scores associated with BMI and WHR respectively in men and women

Models were run to predict BMI and WHR_adjBMI_ according to five different SNP threshold levels of BMI PGS and WHR_adjBMI_ PGS respectively, using independent loci clumped at +/− 250 kb, R2 > 0.25. In the most predictive models that adjusted for age and quality control variables, 3.7% variation in BMI in men was explained by polygenic scores for BMI (derived from *n* = 71 639 SNPs), and in women 4.3% variation in BMI was explained by BMI PGS (*n* = 71 113 SNPs) (see Table S1). In men, 0.6% of the variance in adult WHR (controlling for BMI) was explained by WHR_adjBMI_ PGS (*n* = 53 312 SNPs), and in women 2.2% of the variation in adult WHR was attributable to the WHR_adjBMI_ PGS (*n* = 2281 SNPs) (Tables S2–S5 report equivalent models using three alternative WHR models); these values are highly consistent with reported estimates from meta-analysis^21,38^ ─i.e. 0.46% to 0.8% in men; 1.34% to 2.4% in women. In the total sample, 5.6% of the variation in BMI and 0.8% of the variation in WHR were explained by their equivalent polygenic scores. This percentage variance in BMI is higher than recent reports using BMI-susceptibility loci to explain percentage variance, e.g. 1.5% to 2.7%^19,39,40^. For consistency, polygenic scores used in the main analyses were selected according to the SNP threshold of p < 0.5, which most commonly explained greatest percentage variance in the adiposity phenotype.

### Adiposity PGS associated with moderating factors in men and women

Polygenic scores for BMI were inversely associated with education, income and cognitive ability in men and women respectively, and inversely associated with alcohol intake in women; they were positively associated with material deprivation in men and women respectively and positively associated with physical activity in men (Table S6 for correlation coefficients). Polygenic scores for WHR_adjBMI_ were positively associated with alcohol consumption, material deprivation and neuroticism, and, inversely associated with education and income in men and women respectively; furthermore, in men, they were inversely associated with cognitive ability (Table S6). In all of the above cases these were very small effect sizes (r = −0.04 to 0.03).

### Moderation of genetic susceptibility to adiposity in men and women

**Figure 1:**
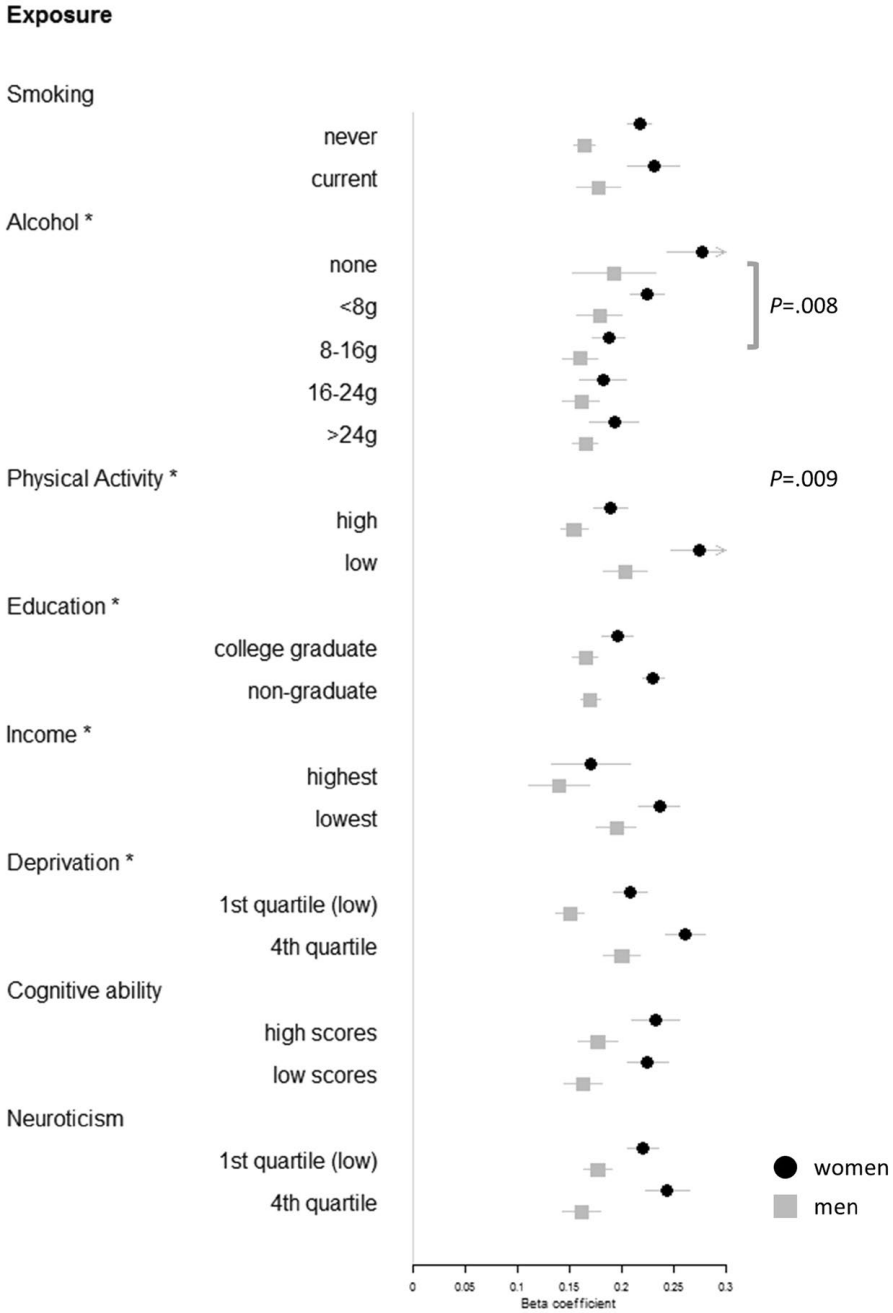
Forest plot showing a one SD change in adult BMI according to a one SD increase in BMI PGS, by strata of lifestyle and psychosocial factors, and by sex. Beta coefficients and 95% confidence intervals derive from models that include age, age^2^, genetic batch, genetic array, assessment centre, and 10 principal components of population structure. Asterisks indicate significant two-way interactions (PGS*Exposure) in predicting BMI in the total sample. P-values show significant three-way interactions with sex (Sex*PGS*Exposure).

Table 2 presents results of the main analyses, which are the *p*-values for interactions between adiposity PGS and each environmental exposure on adiposity phenotypes measured in adulthood (Tables S7 and S8 report the model coefficients). Firstly, these provide evidence that in a UK sample of adults in mid to late adulthood physical activity, education, household income, alcohol consumption, and material deprivation each moderated the effect of genetic susceptibility to overall obesity adulthood (Table S7). Smoking, cognitive ability, and neuroticism were not found to moderate genetic susceptibility for high BMI. Secondly, sex differences were apparent for some of these moderator effects on BMI genetic susceptibility (Table 2; Fig. 1; Table S7). Physical activity (MET mins/week) moderated the effect of BMI PGS to a greater extent in women than in men (Fig. S2a), which was statistically significant in a model of the total sample (p = 0.009; three-way interaction term). Therefore higher levels of physical activity were more strongly related to lower genetic effects on BMI in women relative to men (see Table S12). The moderation effect of non-drinking versus moderate alcohol intake (1–2 units per day) on BMI PGS was significant in women and not men, and this sex difference also reached statistical significance (p = 0.008; three-way interaction). For example, women non-drinkers showed greater genetic susceptibility to elevated BMI (β = 0.278, 95%CI [0.243 to 0.313]) than moderate drinkers (β = 0.188, 95%CI [0.172 to 0.203]); whereas this was also the trend among men, the difference in effects sizes from equivalent models was smaller (β = 0.193, 95%CI [0.153 to 0.233] *vs* β = 0.160, 95%CI [0.143 to 0.177]). The association between BMI PGS and adult BMI was weaker among female college graduates (β = 0.196, 95%CI [0.181 to 0.211]) compared to non-graduates (β = 0.230, 95%CI [0.220 to 0.241], p = 3.89 × 10^−4^), and although this effect was non-significant for men (β = 0.170, 95%CI [0.166 to 0.180] *vs* β = 0.165, 95%CI [0.153 to 0.177], p = 0.474), the sex difference according to three-way interaction in the total sample was non-significant (p = 0.245) (Fig. S2b). High income and low deprivation were each associated with lower genetic manifestation of obesity, and these effects showed no sex differences (Fig. S2b).

**Table 2:** Interaction Term P-values between Polygenic Scores and Environmental Factors in Predicting Adiposity in the Total Sample, and by Sex Group.

*N*	BMI predicted by BMI PGS			WHR predicted by WHR_adjBMI_ PGS		
	Total 111 819	Men 53 065	Women 58 754	Total 111 956	Men 53 147	Women 58 809
*Smoking*						
current vs never	0.354	0.685	0.911	0.170	0.238	0.994
previous vs never	0.481	0.068	0.040	0.147	0.668	0.881
*Alcohol*						
gpd^a^	0.132	0.376	0.440	0.035	0.722	0.331
none vs 8–16 g	**5.34 × 10** ^**−12**^	0.060	**8.44 × 10** ^**−10**^	0.069	0.893	0.265
<8 g vs 8–16 g	**1.71 × 10** ^**−4**^	0.156	**0.004**	0.099	0.835	0.848
>24 g vs 8–16 g	0.611	0.992	0.746	0.174	0.581	0.906
16–24 g vs 8–16 g	0.071	0.695	0.844	0.065	0.857	0.401
*Physical activity*						
MET mins/week^b^	**2.19 × 10–14**	**0.002**	**2.06 × 10** ^**−9**^	0.067	0.082	0.137
moderate vs low	**1.03 × 10** ^**−11**^	**0.003**	**4.03 × 10** ^**−6**^	0.320	0.786	0.834
high vs low	**6.35 × 10** ^**−18**^	**7.31 × 10** ^**−5**^	**4.19 × 10** ^**−10**^	0.029	0.091	0.422
*Education*						
graduate vs non	**6.33 × 10** ^**−5**^	0.474	**3.89 × 10** ^**−4**^	0.353	0.890	0.085
*Income*						
mean income	**1.19 × 10** ^**−10**^	**0.002**	**0.010**	0.007	0.582	**7.40 × 10** ^**−4**^
£18–£31 k vs <£18 k	0.004	0.023	0.785	0.006	0.069	0.197
£31–£52 k vs <£18 k	**2.21 × 10** ^**−**^ **4**	0.115	0.209	**0.002**	0.769	0.020
£52–£100 k vs <£18 k	**4.61 × 10** ^**−8**^	**0.002**	0.116	**0.004**	0.356	**0.002**
>£100 k vs <£18 k	**6.76 × 10** ^**−7**^	**0.006**	**0.017**	0.132	0.293	0.093
*Deprivation*						
Townsend score	**7.62 × 10** ^**−14**^	**7.06 × 10** ^**−7**^	**5.60 × 10** ^**−8**^	0.031	0.066	0.033
2^nd^ quartile vs 1st	0.398	0.241	0.747	0.109	0.912	0.188
3^rd^ quartile vs 1st	**0.002**	**0.015**	0.543	0.030	0.844	0.004
4^th^ quartile vs 1st	**2.36 × 10** ^**−9**^	**2.43 × 10** ^**−6**^	**9.19 × 10** ^**−6**^	0.013	0.286	0.007
*Cognitive ability*						
score	0.787	0.196	0.641	0.285	0.824	0.596
*Neuroticism*						
score	0.058	0.543	0.085	**0.003**	0.345	0.119
2^nd^ quartile vs 1st	0.700	0.162	0.813	0.784	0.138	0.664
3^rd^ quartile vs 1st	0.809	0.469	0.873	0.863	0.809	0.696
4^th^ quartile vs 1st	0.209	0.139	0.055	**0.003**	0.270	0.138

Statistically significant p-values in bold survive correction for multiple testing according to FDR (p ≤ 0.017 for 78 BMI models; p ≤ 0.004 for 78 WHR models). For the total sample, significant 3-way interaction terms for Sex*BMI PGS*[Exposure] were observed for Alcohol (none vs 8–16 g): p = 0.008, and Physical activity (MET mins/week): p = 0.009 (see Tables S7 and S8 for coefficients of these models). ^a^Model includes drinkers of 1 unit (8 g) or more per day. ^b^Square-root value used for normality.

**Figure 2:**
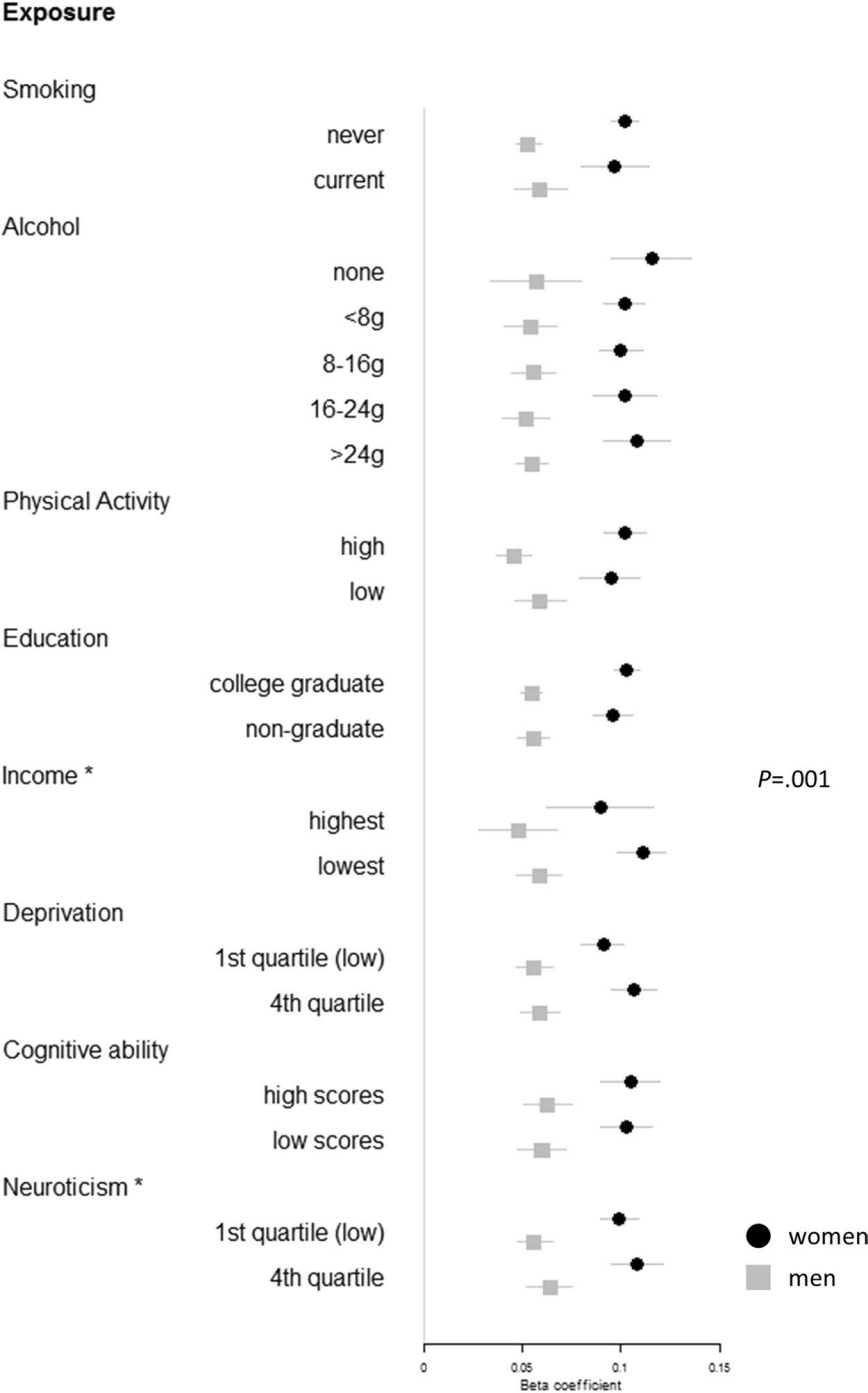
Forest plot showing a one SD change in adult WHR according to a one SD increase in WHR_adjBMI_ PGS, by strata of lifestyle and psychosocial factors, and by sex. Beta coefficients and 95% confidence intervals derive from models that include age, age^2^, BMI, genetic batch, genetic array, assessment centre, and 10 principal components of population structure. Asterisks indicate significant two-way interactions (PGS*Exposure) in predicting WHR in the total sample. P-value shows significant three-way interaction with sex (Sex*PGS*Income).

In contrast to BMI models, manifestation of genetic risk of abdominal obesity (WHR) was moderated by income and neuroticism, but not smoking, alcohol intake, physical activity, education, material deprivation, nor cognitive ability (see Table 2, Fig. 2, and Table S8 for main results of models that include WHR_adjBMI_ PGS as the exposure, and BMI as a covariate; see Tables S9–11 for variations of WHR models where BMI is not accounted for). Among participants within relatively higher household income bands the associations between WHR_adjBMI_ and WHR phenotype were considerably weaker relative to those in lower income bands (Table S13; Fig. S3a–c). This moderation effect was observed in women (£52–100 k per year: β = 0.086, 95%CI [0.073 to 0.100]; <£18 k: β = 0.111, 95%CI [0.098 to 0.123], p = 0.002) and not men (β = 0.054, 95%CI [0.044 to 0.064] *vs* β = 0.059, 95%CI [0.047 to 0.070], p = 0.006), although the sex difference failed to reach statistical significance in a three-way interaction term corrected for multiple testing (p = 0.001). Furthermore, the association between WHR_adjBMI_ PGS and WHR was also reduced among participants scoring low in neuroticism in the total sample (lowest quartile: β = 0.086, 95%CI [0.078 to 0.093]; highest quartile: β = 0.099, 95%CI [0.091 to 0.107], p = 0.004), and there were no apparent sex differences.

### Quantile interpretation of sex differential effects

Among women within the highest risk decile for BMI PGS low physical activity was associated with a 2.63 kg/m^2^ higher BMI on average relative to high physical activity, whereas among women in the lowest decile group the difference was 1.26 kg/m^2^ in the lowest decile group (Fig. S2a). In men, the equivalent values were 1.68 kg/m^2^ and 0.95 kg/m^2^.

The interaction effect of alcohol intake on genetic risk for high BMI in women relative to men, was driven by women who drank <1 unit per day having a greater risk of high BMI according to their polygenic risk score, compared to those who drank more (Tables 2; S6 and S12). Among women within the highest decile for BMI PGS non-drinkers relative to those who drank on average 1–2 units per day showed a 2.71 kg/m^2^ higher BMI on average, whereas among women in the lowest decile for polygenic risk abstainers were on average 0.87 kg/m^2^ higher in BMI than moderate drinkers (Fig. S2a). In men the equivalent differences were 0.39 kg/m^2^ and 0.17 kg/m^2^.

We translate the moderating effect of college education on manifestation of genetic risk for overall obesity that was evident in women but not men. Among women within the highest decile for BMI PGS, being a non-graduate was associated with a 1.75 kg/m^2^ higher BMI compared to a graduate, whereas for women within the lowest decile for BMI PGS the mean difference between non-graduates and graduates was 1.03 kg/m^2^ (Fig. S2b). The equivalent differences among men were 1.19 kg/m^2^ and 1.09 kg/m^2^ respectively.

Among women within the highest decile for WHR_adjBMI_ PGS an average annual household income of <£18,000 relative to £52–100,000, was associated with a 0.032-point higher WHR on average, compared to a difference of 0.024 in the lowest decile group for genetic susceptibility (Fig. S3a). In men, the equivalent values were 0.031 and 0.035 respectively.

### Sensitivity analyses

In post-hoc analyses we considered sex-differential effects within three separate age groups of the cohort (Table S14 reports descriptive data for each exposure by age group). Firstly, we found that the stronger effect in women of physical activity on genetic influences on BMI was observable in those in their forties, but not within older age bands: 40–49 year-olds only (3-way interaction term: *p* = 0.022); among 50–59 year-olds (*p* = 0.39) or 60–73 year-olds (*p* = 0.26) (Fig. S4). Secondly, the greater moderating effect of alcohol consumption (none vs 8–16 g) in women relative to men on genetic susceptibility to high BMI was significant among 50–59 year-olds (*p* = 0.001), but not 40–49 years old (*p* = 0.58) and 60–73 year-olds (*p* = 0.10) (Fig. S5). Thirdly, the greater effect of income among women on genetic susceptibility to central obesity was significant among 40–49 years olds (p = 0.026) and 50–59 year-olds (p = 0.02), but not among or 60–73 year olds (p = 0.22) (Fig. S6).

## Discussion

The present study considered potential moderation by several lifestyle and psychosocial factors on manifestation of genetic susceptibility to two adiposity indicators, with a focus on sex-specific effects, using UK Biobank baseline data. There was a clear difference in the range of these factors that interacted with polygenic scores for BMI and WHR_adjBMI_ respectively. In the total sample, genetic influence on adult BMI was statistically attenuated by higher physical activity, moderate versus no alcohol consumption, higher education, higher income and lower material deprivation, respectively. No interaction effects were observed for smoking, cognitive ability, and neuroticism. In contrast, for models predicting WHR, higher household income and low *vs* high neuroticism were associated with an attenuation of the WHR_adjBMI_ polygenic score effect. The present study also exposed some notable sex differences in these interactions. The influence of greater physical activity in attenuating the polygenic effect on BMI was greater in women than men. The interactions of alcohol consumption and college education with BMI PGS were evident in women but not men, albeit the sex difference was significant for alcohol only. Finally, higher income influenced the polygenic effects on WHR in women, but not men.

Numerous studies have reported on the moderating effects of physical activity on manifestation of genetic susceptibility to obesity, according to either single^10–12,15–17^ or several candidate genes for BMI^8,13,14,41^. However, we are aware of only one study that tested sex-specific effects of physical activity (active *vs* inactive) and genetic loci on adiposity (BMI and WHR_adjBMI_ respectively), which reported no significant genome-wide interactions for the total sample (except *FTO* gene) or sex-specific groups^41^. Whereas we observed that physical activity was a considerable moderator for both sexes, the magnitude of its effect in attenuating genetic risk of elevated BMI was greater among women than men (p = 0.009), when physical activity was a continuous measure of MET/mins per week, rather than stratified. The effect was only significant among 40 to 49-year-olds and not among older age bands, which may be explained by stronger genetic associations on adult BMI observed in ≤50-year-olds, compared to older adults^42^. We consider these sex and age effects important regardless of the underlying mechanisms that explain the observed interaction between physical activity and genetic effects on BMI; for example, whether they are used to select appropriate groups for clinical intervention (if physical activity is a true moderator of genetic obesity), or equally, whether they help us appropriately account for sex differences in self-report bias, to fully account for genetic effects on BMI variance. Data from objective measures of physical activity, i.e. accelerometers, available in future releases of UK Biobank data^43^, could be utilised to test out this source of bias.

Moderate daily alcohol consumption (estimated from self-reported weekly intakes of various wines, beers and spirits) versus abstinence similarly showed stronger interaction effects with BMI PGS for women (p = 0.008). If comparing the results of women who reported drinking moderately (8 to 16 grams/day) with their non-drinking peers their genetic susceptibility to high BMI was attenuated by ~30%, compared to about half that for men. There were however no differences in genetically-influenced BMI between moderate consumers and those who drank more, in the total group or men and women respectively. It is possible that an underlying biological mechanism may yet explain the interaction effect of alcohol in women. Alternatively, it could be that women in UK Biobank will have modified their alcohol intake differently to men, for example, by becoming abstainers in response to genetically-determined obesity or comorbid health conditions prior to the study’s baseline – i.e. FTO carriers are shown to have lower alcohol frequency^10,44^. However, one previous study showed that accounting for self-reported changes in alcohol frequency over ten years made no change to the interaction between alcohol and FTO in predicting BMI^10^. Otherwise, it is possible, as alluded to above, that self-report bias acts differently for men and women. Whereas previous studies have reported on stronger genetic susceptibility to obesity in non-drinkers versus moderate or high-level drinkers—either according to consumption levels^20^ as in the present study, or frequency^8,19^ —this is the first study perhaps to show the effect to be prominent in women but not men. The message that moderate alcohol intake is good news for genetic susceptibility to BMI compared to non-drinking resonates with a research literature that supports a cardio-protective role of low-to-moderate levels of alcohol intake^45,46^. However, a recent meta-analysis has challenged this well-documented effect, with evidence that it may be due to selection biases in older age cohorts^47^.

Relatively fewer studies have considered psychosocial factors as moderators of genetic influence on adiposity. Perhaps unsurprisingly we observed the same strong interaction effects for Townsend (material) deprivation^8,19^ and income^19^ reported by recent studies of UK Biobank, albeit these used pre-selected genetic loci for BMI. Additionally, we report the novel finding that college graduates showed attenuated genetic effects on obesity relative to their non-graduate peers. Our findings of interaction by educational attainment may depart from the afore-mentioned studies, due to differences in measurement of education, and/or, BMI genetic risk. Evidence from the Health and Retirement Study previously reported interaction effects between parental education and the BMI-related SNP *rs9540493* on BMI^48^. Although in such cases education may be acting as a proxy for differences in early socioeconomic differences, a recent study of UK Biobank reported highly significant genetic correlations between educational attainment and BMI^49^, which could suggest that our results for education are independent, and, biologically plausible. To support this, new evidence from a natural experimental study of the same cohort, which exploited variance in educational attainment resulting from a UK government reform in compulsory schooling age in 1972, showed a causal association between the age of leaving secondary education and midlife obesity that was moderated by BMI PGS, i.e. the association between PGS for BMI and obesity was less strong in those who left education later^50^. We observed the moderation effect of college education to be significant in women and not men (although the p-value for this sex difference did not survive correction for multiple testing), and women with more education are found to more readily modify their behaviour in response to health issues relative to their male counterparts^51^, which could have influenced this result.

An important aspect of the present study was to consider moderator effects on genetic susceptibility to abdominal obesity compared to those of total obesity, given their distinct genetic make-up^42^. Genetic susceptibility to abdominal obesity (i.e. WHR) was not found to be moderated by as many exposures in the present study, which may be related to lower statistical power given that the main effects of WHR_adjBMI_ PGS on WHR phenotype were smaller to those for BMI PGS on BMI. Whereas material deprivation was the more potent moderator of genetic susceptibility to BMI compared to income, income was the single socioeconomic status indicator to influence genetic effect on WHR. This effect was again observed in women only. In a recent meta-analysis 44 sex-specific loci were identified for their association with WHR^42^, 28 of which were stronger in women, which is likely to have contributed to our sex differential effect. A second novel finding that we observed for gene-environment effects on abdominal obesity, was the significant interaction by the personality trait, neuroticism. High neuroticism, which has previously been associated with increased risk of elevated BMI^29^, was found to inflate manifestation of genetic susceptibility to WHR. This was apparent when comparing those in the highest relative to lowest quartile on this self-rated trait measure. Evidence from UK Biobank shows neuroticism to be a highly polygenic trait^52^, exhibiting pleiotropy with co-morbidities of central obesity including depression, coronary heart disease, and smoking^53^, so there is the potential for genetics influencing this effect. Whereas higher pre-morbid cognitive ability has previously been associated with reduced obesity risk in a UK-population based cohort^30^, and has shown to moderate the manifestation of genetic susceptibility to type 2 diabetes^32^, we found no evidence that cognitive ability was an important moderator in our models of the total sample or by sex group.

Lifestyle and psychosocial indicators were analysed contemporaneously of adiposity measures in the present study, and so we cannot rule out likelihood of their earlier modification due to changes in BMI/WHR; this would have acted to inflate the observed interaction effects if any such changes were caused by genetic influence on BMI/WHR accounted for by the polygenic scores. For example, evidence suggests that individuals may lessen their alcohol consumption following an increase in their BMI status^10^. Very few studies have enabled consideration of interaction effects of genes and lifestyle on later adiposity (as opposed to cross-sectional analyses). However, at least one reported that the moderating effect of physical activity was seen for associations between genetic influences on BMI and change in BMI over the next decade^18^. Additionally, our cross-sectional data failed to consider cumulative load of protective or adverse lifestyle habits. Whereas psychosocial factors such as education, material deprivation, and neuroticism, are fairly stable from early adulthood to midlife─i.e. the baseline age of UK Biobank, behaviours such as alcohol consumption and physical activity are more variable over the life course. Future follow-up of this cohort will enable consideration of changes in lifestyle behaviours and their cumulative load over time, in respect of their moderation on genetic influences. Another weakness of the present study is that the sex differential effects observed for physical activity and alcohol consumption moderating genetic susceptibility to obesity may have been subject to self-report bias, in ways that may have either supressed or inflated our findings, and we were unable to test for this. Finally, our findings are generalizable to UK residents of European ancestry but not to populations who may vary in their genetic susceptibilities to adiposity and/or their lifestyle exposures─particularly their sex–specific nature.

A main strength of the present study is its assessment of two adiposity measures, specifically the inclusion of WHR to indicate body fat distribution, which is a greater predictor of health outcomes than BMI when measured after mid-adulthood. We not only provide novel evidence that specific lifestyle and psychosocial factors statistically moderate manifestation of genetic susceptibility to central adiposity as well as total adiposity, but we have exposed differences in the effects for specific moderators for these two outcomes. For example, we found evidence that neuroticism is a potential moderator of the manifestation of genetic susceptibility to abdominal but not total obesity. A second main strength of the present study is our employment of genome-wide polygenic scores, which explained over twice as much variance in BMI than the majority of previous studies using pre-selected genetic loci^8,19,40^. These scores incorporating genotyped data from vast numbers of SNPs, may have increased the opportunity to detect gene-exposure interaction effects, relative to studies that use pre-selected genetic for their significant main effects on adiposity.

Any composite score of genetic risk for a complex phenotype might mask some gene-environment interactions if variants are not all associated with the phenotype in the same direction. A recent meta-analysis specifically reported on interaction effects of smoking with individual SNPs in the prediction of adiposity phenotypes, identifying two loci for BMI and five for WHR^54^. All WHR-smoking loci were listed in the meta-analysis of significant hits from which we derived our WHR_adjBMI_ polygenic scores^38^. However, neither of the BMI-smoking loci were included in the meta-analysis of significant hits from which we derived our BMI PGS^40^. By missing potentially important variants identified through meta-analyses of interaction effects we may have reduced our chances of detecting effects for smoking, reported by previous studies^18,19,55^.

In summary, in a large UK-based population cohort of men and women, we have found evidence for the moderating effects of several psychosocial factors on genetic influence on adiposity; that is, higher education, higher income, and lower material deprivation attenuate associations between BMI polygenic scores and adult BMI, and, higher income and lower neuroticism attenuate genetic susceptibility to abdominal obesity. Additionally, we replicate findings that physical activity and alcohol consumption moderate genetic influences on adiposity. Crucially, we demonstrate the importance of considering sex-specific effects in studying gene-by-environment interactions in complex diseases.

## Methods

### Background

The UK Biobank health resource provided the data for the present study. It is a prospective cohort study of >500,000 men and women, aged 40 to 73 years when they were recruited between 2007 and 2010, and represents 22 geographical regions of the UK^56^. At baseline the participants provided blood samples, had impedance measures taken by a trained nurse, and completed a touchscreen assessment including questions on lifestyle and psychosocial factors, and a cognitive assessment test battery. These data were used in the current analyses, which are restricted to approximately one third of the total sample, for which genotyping data were first released.

### Anthropometric measures

Measurements were recorded by a trained nurse for weight in kilograms using a bioelectrical impedance device ‘Tanita BC418MA’, and standing height in metres using a Seca 240 cm height measure. These were converted to body mass index (weight/height^2^). Waist and hip circumference (cm) were taken at the same time using a Seca 200 cm tape measure, and converted to WHR values (waist/hip). Full details of the anthropometric assessment are available online^57^. In the present study’s analytic sample 53,065 men and 58,754 had BMI measures and 53,147 men and 58,809 women had WHR measures.

### Genotyping

The interim release of UK Biobank included genotype data for 152,729 individuals, of whom 49,979 were genotyped using the UK BiLEVE array and 102,750 using the UK Biobank axiom array. These arrays have over 95% content in common. Quality control included removal of participants based on missingness, relatedness, gender mismatch, and non-British ancestry. Details of the array design, genotyping and quality control procedures have been published elsewhere^49,58^. Variants with a minor allele frequency of less than 0.01 and non-autosomal variants were excluded from further analysis.

### Polygenic scores

PRSice^59^ was used to derive polygenic risk scores using data from the GIANT consortium European-only meta-analysis for BMI^40^ and waist-to-hip ratio with and without adjustment for BMI (WHR and WHR_adjBMI_)^38^. PRSice calculates the sum of alleles associated with the phenotype of interest (BMI and WHRadjBMI) across many genetic loci, weighted by their effect sizes estimated from a GWAS of the corresponding phenotype in an independent sample. The GWAS summary statistics for BMI, WHR and WHRadjBMI were used as the training (base) dataset, while UK Biobank was used as the prediction (target) dataset. Specifically, polygenic risk scores were created for BMI, BMI (female only), BMI (male only), WHRadjBMI, WHRadjBMI (female only), WHRadjBMI (male only) (and equivalent for WHR). Single nucleotide polymorphisms (SNPs) with a minor allele frequency below 0.01 were removed prior to creating the scores. Independent SNPs in linkage disequilibrium were obtained by clumping with an r^2^ < 0.25 within a +/− 250 kb window. The distance threshold of 250 kb is the default value for clumping procedures in PRSice. Whereas we acknowledge that the 250 kb threshold for clumping might not be optimal for all loci ─ given that multiple SNPs may then be included that show an association based on the same independent SNP ─ there is the possibility that independent SNPs could be excluded if the threshold was to be increased^60^. For consistency across the different polygenic risk scores and loci we used the default value of 250 kb set by PRSice. This clumps any SNP that is within 250 kb to both ends of the index SNP, meaning that each locus is 500 kb in length. The scores were calculated according to multiple SNPs that had significance values in predicting the anthropometric measures, of p < 1.0, p < 0.5, p < 0.1, p < 0.05, and, p < 0.01 respectively.

### Psychosocial and lifestyle factors

Psychosocial and health behaviour data were recorded via computer touchscreen at the UK Biobank Assessment Centre between April 2007 and July 2010. Lifestyle factors selected for potential moderation of genetic susceptibility to adiposity included smoking, alcohol consumption, and physical activity. Smoking habits in the present study were confined to a three-level categorical variable, according to whether participants: never smoked, were ex-smokers, or were current smokers. The intention to ascertain a dosage measure of tobacco use was rejected due to the relatively small number of current smokers who completed these questionnaire items. Dosage of alcohol consumption, according to *n* grams per day, was estimated from a reduction of multiple items – described elsewhere^61^ - on which the majority of participants responded, including: (i) frequency of alcohol drinking, (ii) consumption per type of alcohol drank per week and/or month. We used this continuous measure to derive a five-level categorical variable reflecting UK alcohol unit measures: abstainers (0 units), <8 g (<1 unit), 8–16 g, 16–24 g, and >24 g per day. Physical activity was assessed according to items from the International Physical Activity Questionnaire (IPAQ), and reduced to a single continuous measure of MET (Metabolic Equivalent of Task) minutes per week, for which we derived its square root-value to normalise the distribution. A categorical scale was created of low, medium, and high levels of physical activity according to IPAQ recommendations^62^.

Among the psychosocial measures included as potential moderators were educational attainment, household income, and material deprivation. In the present study educational attainment was indicated dichotomously: whether someone was a college graduate or not. Average gross household income was measured according to five categories, and included as continuous and categorical variables in the analyses: <£18k, £18 to 30,999, £31k to £51,999, £52k to £100 K, and, >£100,000. Material deprivation was continuously measured by the Townsend Deprivation Index^63^ according to individuals’ postcode location. The index uses updated census data on unemployment, non-car and non-home ownership, and house overcrowding, deriving a standardised score, whereby 0 equates to average national deprivation, a positive score indicates greater material deprivation, and a negative score relative affluence. We further analysed deprivation as a categorical variable by quartiles. Finally, two psychological traits were considered as potential psychosocial moderators in the present study due to their reported associations with obesity, metabolic syndrome, and CVD: cognitive ability, and the personality trait of neuroticism—well-validated as reflecting stable individual differences in self-reported ratings of negative emotions^64^. General cognitive ability was indicated according to responses on 13 multiple-choice items of verbal and numerical reasoning, within a maximum two-minute period, which has previously shown good longitudinal stability in this cohort^65^. A neuroticism score was calculated from responses to the 12 items of the Eysenck Personality Questionnaire (EPQ-R)-Short Form─a score of 12 indicated the highest level of neuroticism. Due to its curvilinear association with BMI (Fig. S2b) we analysed neuroticism according to quartile groups.

### Statistical analyses

Anthropometric measures and PGS were centred and standardised for analytic purposes. In BMI prediction models, cases were excluded if a BMI value was considered implausible (<14.9 kg/m^2^ or >60 kg/m^2^; n = 21). In WHR prediction models, cases were excluded if WHR was >4 SDs beyond the mean in either direction (e.g. <0.513 or >1.238; n = 16). To optimise the statistical power of our models we used continuous measures of environmental exposures wherever possible. Where continuous exposures reached statistical significance in their interaction with polygenic scores, we repeated the models using categorical variables of the moderators─ by median split or interquartile range in the absence of pre-defined groups─in order to obtain results that could be displayed and interpreted.

Basic linear models were run consecutively to predict the anthropometric outcome (BMI, WHR or WHR_adjBMI_) according to each of five polygenic scores (i.e. estimated according to five different significance thresholds in the GWAS summary data); whichever SNP threshold for a polygenic score most often explained greatest percentage variance in the adiposity phenotype, was selected for all subsequent models. Linearity of bivariate associations between PGS and continuous environmental exposures were assessed by plotting regression curves that allowed for restricted cubic splines (included in Supplementary Material); otherwise, for categorical lifestyle or psychosocial exposures we compared mean PGS by group using t-tests or ANOVA.

Age and age^2^-adjusted (and sex-adjusted for total sample) linear regression models were used to predict adult BMI or WHR, with a marginal term for the PGS and environmental exposure respectively, and a two-way interaction term for PGS*[environmental exposure]. All models included additional covariates to adjust for quality control (batch, array, and assessment centre) and population structure of the genetic data (the first 10 principal components – see Supplementary Material for an explanation of these covariates). For prediction of WHR, the main results report on models that include WHR_adjBMI_ PGS as the exposure and BMI phenotype as a covariate, and variations on these are included in the supplementary file (Table S9 - WHR PGS including BMI as covariate; Table S10 - WHR_adjBMI_ PGS without BMI; Table S11 - WHR PGS without BMI). For subsequent models, in which we tested sex differences of the moderator effects, we included the following three interaction terms: (i) two-way sex*PGS; (ii) two-way sex*[environmental exposure]; (iii) 3-way sex*PGS*[environmental exposure]. R was used to test for the false discovery rate (FDR)^66^ to indicate those model terms that retained statistical significance after taking account of multiple testing. From the main analyses (Table 2) *p*-values of 78 terms among 39 models for BMI and WHR respectively were included in FDR correction. Otherwise, analyses were conducted using STATA version 14.

## Electronic supplementary material

Supplementary materials

## Data Availability

The third-party data that support the findings of this study are available from UK Biobank, which is an open access resource for international bona fide researchers (http://www.ukbiobank.ac.uk/register-apply/). The authors do not have permission to distribute the data themselves.
