# Supplementary material for: Sex-specific moderation by lifestyle and psychosocial factors on the genetic contributions to adiposity in 112,151 individuals from UK Biobank
Source: Sci Rep. 2019 Jan 23;9:363. doi: 10.1038/s41598-018-36629-0 (PMC6344557; doi:10.1038/s41598-018-36629-0)

Sex-specific moderation by lifestyle and psychosocial factors on the genetic contributions to adiposity in 112,151 individuals from UK Biobank

Catherine M. Calvin, Saskia P Hagenaars, John Gallacher, Sarah E Harris, Gail Davies, David C. Liewald, Catharine R Gale, and Ian J. Deary

Supplementary Information

S1 Table

Associations between BMI PGS and adult BMI according to five different SNP thresholds in men (n=53 063) and women (n=58 751) respectively. Models are adjusted for age, age^2, genetic batch, genetic array, assessment centre, and 10 principal components of population structure.

**Sex**	**BMI PGS Threshold**	**SNPs, *n***	**β**	**95% CI**	**R2**
**Women**	0.01	2229	0.188	0.180, 0.197	0.0303
	0.05	8286	0.209	0.200, 0.217	0.0372
	0.1	15 527	0.213	0.204, 0.221	0.0388
	0.5	71 113	0.224	0.216, 0.233	0.0433
	1	128 441	0.225	0.216, 0.233	0.0432
**Men**	0.01	2297	0.144	0.137, 0.152	0.0259
	0.05	8565	0.163	0.155, 0.170	0.0330
	0.1	16 088	0.170	0.162, 0.177	0.0357
	0.5	71 639	0.172	0.165, 0.180	0.0369
	1	126 975	0.173	0.165, 0.180	0.0371

S2 Table

Associations between WHR_{adjBMI} PGS and adult WHR according to five different SNP thresholds in men (n=53 033) and women (n=58 725) respectively.

Models are adjusted for age, age^2, BMI, genetic batch, genetic array, assessment centre, and 10 principal components of population structure.

**Sex**	**WHR PGS Threshold**	**SNPs, *n***	**β**	**95% CI**	**R2**
Women	0.01	2281	0.114	0.109, 0.120	0.0215
	0.05	8735	0.107	0.102, 0.113	0.0188
	0.1	17 081	0.107	0.102, 0.112	0.0185
	0.5	72 819	0.102	0.097, 0.108	0.0166
	1	126 094	0.100	0.095, 0.106	0.0161
Men	0.01	1633	0.045	0.040, 0.050	0.0039
	0.05	6618	0.051	0.046, 0.056	0.0050
	0.1	12 965	0.055	0.050, 0.059	0.0057
	0.5	53 312	0.056	0.052, 0.061	0.0060
	1	90 087	0.057	0.052, 0.062	0.0062

S3 Table

Associations between WHR PGS and adult WHR according to five different SNP thresholds in men (n=53 033) and women (n=58 725) respectively. Models are adjusted for age, age^2, BMI, genetic batch, genetic array, assessment centre, and 10 principal components of population structure.

**Sex**	**WHR PGS Threshold**	**SNPs, *n***	**β**	**95% CI**	**R2**
Women	0.01	2117	0.089	0.084, 0.095	0.0131
	0.05	8587	0.088	0.082, 0.093	0.0126
	0.1	16 837	0.088	0.082, 0.093	0.0124
	0.5	72 091	0.083	0.078, 0.089	0.0110
	1	125 514	0.083	0.077, 0.088	0.0109
Men	0.01	1636	0.028	0.023, 0.032	0.0015
	0.05	6747	0.032	0.027, 0.037	0.0020
	0.1	13 489	0.038	0.033, 0.043	0.0028
	0.5	56 245	0.039	0.034, 0.044	0.0029
	1	95 401	0.039	0.034, 0.044	0.0029

S4 Table

Associations between WHR_{adjBMI} PGS and adult WHR according to five different SNP thresholds in men (n=53 145) and women (n=58 806) respectively. Models are adjusted for age, age^2, genetic batch, genetic array, assessment centre, and 10 principal components of population structure. BMI is not included as a covariate.

**Sex**	**WHR PGS Threshold**	**SNPs, *n***	**β**	**95% CI**	**R2**
Women	0.01	2281	0.108	0.102, 0.114	0.0192
	0.05	8735	0.104	0.098, 0.111	0.0177
	0.1	17 081	0.104	0.098, 0.110	0.0174
	0.5	72 819	0.098	0.092, 0.105	0.0154
	1	126 094	0.097	0.090, 0.103	0.0149
Men	0.01	1633	0.043	0.037, 0.049	0.0034
	0.05	6618	0.051	0.045, 0.057	0.0049
	0.1	12 965	0.054	0.048, 0.060	0.0056
	0.5	53 312	0.057	0.051, 0.063	0.0062
	1	90 087	0.058	0.052, 0.064	0.0064

S5 Table

Associations between WHR PGS and adult WHR according to five different SNP thresholds in men (n=53 145) and women (n=58 806) respectively. Models are adjusted for age, age^2, genetic batch, genetic array, assessment centre, and 10 principal components of population structure. BMI is not included as a covariate.

**Sex**	**WHR PGS Threshold**	**SNPs, *n***	**β**	**95% CI**	**R2**
Women	0.01	2117	0.102	0.096, 0.108	0.0172
	0.05	8587	0.106	0.100, 0.112	0.0183
	0.1	16 837	0.109	0.103, 0.115	0.0192
	0.5	72 091	0.107	0.101, 0.113	0.0184
	1	125 514	0.108	0.101, 0.114	0.0184
Men	0.01	1636	0.058	0.052, 0.064	0.0064
	0.05	6747	0.068	0.062, 0.074	0.0087
	0.1	13 489	0.077	0.071, 0.083	0.0113
	0.5	56 245	0.081	0.075, 0.087	0.0123
	1	95 401	0.081	0.075, 0.087	0.0124

S6 Table

Pearson correlation coefficients between polygenic risk scores, weight indices, and environmental factors, in women (upper diagonal) and men (lower)

	**BMI PGS**	**WHR_{adjBMI} PGS**	**BMI, kg/m^2**	**WHR**	**Alcohol, gpd**	**Physical activity**	**Education years**	**Income**	**Deprivation**	**Cognitive ability**	**Neurot.**
BMI PGS		-	.21	.07	-.01	-	-.04	-.03	.03	-.03	-
WHR PGS_{adjBMI}	-.02		-	.13	.02	-	-.02	-.01	.02	-	.01
BMI, kg/m^2	.19	-		.46	-.03	-.10	-.14	-.13	.11	-.05	.03
WHR	.10	.08	.60		.05	-.05	-.15	-.18	.11	-.08	.04
Alcohol, gpd	-	.02	.07	.08		-	.05	.09	.04	.06	.03
Physical activity	.02	-	-.06	-.08	.04		-.08	-.08	.02	-.12	-.04
Educational years	-.04	-.04	-.15	-.20	-.06	-.19		.40	-.11	.41	-.10
Income	-.01	-.02	-.06	-.17	.03	-.12	.43		-.22	.25	-.09
Deprivation	.01	.02	.05	.09	.06	.06	-.17	-.26		-.09	.08
Cognitive ability	-.02	-.02	-.06	-.09	.04	-.16	.44	.30	-.12		-.07
Neuroticism	-	.02	.01	.04	.02	-.02	-.06	-.09	.10	-.04	

Coefficients are shown for those statistically significant after correction for multiple testing (FDR; at $p \leq .0372$).

S7 Table

Models predicting BMI from BMI PGS, lifestyle and psychosocial factors, and their two-way interactions (in total sample, men, and women), and additionally, three-way interactions with sex (total sample)

Model		Total N (men, n)	Total sample		3-way term with sex (m)		Men		Women	
			β (SE)	P	*β* (SE)	P	*β* (SE)	P	*β* (SE)	P
**1.**	**Basic model**	111 814								
	BMI PGS	(53 063)	0.236 (0.003)				0.172 (0.004)		0.224 (0.004)	
**2.**	**Smoking**	111 520								
	BMI PGS	(52 930)	0.235 (0.004)				0.166 (0.005)		0.218 (0.006)	
	Smoking status (never, previous, current)		0.026 (0.004)				0.034 (0.005)		0.026 (0.006)	
	*PGS*Smoking (previous vs never)*		0.004 (0.006)	.481	-0.014 (0.013)	.254	0.015 (0.008)	.068	0.020 (0.010)	.040
	*PGS*Smoking (current vs never)*		-0.008 (0.009)	.354	-0.007 (0.018)	.684	0.005 (0.012)	.685	-0.002 (0.014)	.911
**3.**	**Alcohol**	61 362								
i.	BMI PGS	(34 864)	0.213 (0.007)				0.155 (0.009)		0.180 (0.012)	
	Alcohol intake, gpd		0.003 (0.000)				0.004 (0.000)		0.003 (0.001)	
	*PGS*Alcohol intake*		-0.000 (0.000)	.132	-0.000 (0.001)	.671	0.000 (0.000)	.376	0.000 (0.001)	.440
		91 045								
ii.	BMI PGS	(44 350)	0.213 (0.006)				0.160 (0.009)		0.190 (0.009)	
	Alcohol intake, 5-levels		0.014 (0.002)				0.024 (0.003)		0.002 (0.003)	
	*PGS*Alcohol intake (none vs 8-16g)*		0.084 (0.012)	**$5.34e^{-12}$**	-0.066 (0.025)	**.008**	0.035 (0.018)	.060	0.104 (0.017)	**$8.44e^{-10}$**
	*PGS*Alcohol intake (<8g vs 8-16g)*		0.033 (0.009)	**$1.71e^{-04}$**	-0.022 (0.018)	.226	0.019 (0.014)	.156	0.034 (0.012)	**.004**
	*PGS*Alcohol intake (>24g vs 8-16g)*		-0.005 (0.010)	.611	0.000 (0.020)	.985	0.000 (0.013)	.992	-0.005 (0.015)	.746
	*PGS*Alcohol intake (16-24g vs 8-16g)*		-0.015 (0.009)	.071	-0.002 (0.018)	.901	0.004 (0.011)	.695	0.003 (0.015)	.844
**4.**	**Physical activity**	87 337								
i.	BMI PGS	(43 539)	0.273 (0.006)				0.194 (0.008)		0.273 (0.010)	
	Physical activity, METS mins/week		-0.004 (0.000)				-0.003 (0.000)		-0.006 (0.000)	
	*PGS*Physical activity*		-0.001 (0.000)	**$2.19e^{-14}$**	0.000 (0.000)	**.009**	-0.000 (0.000)	**.002**	-0.001 (0.000)	**$2.06e^{-09}$**
		87 337								
ii.	BMI PGS	(43 539)	0.284 (0.007)				0.203 (0.010)		0.278 (0.011)	
	Physical activity groups		-0.176 (0.005)				-0.140 (0.006)		-0.219 (0.007)	
	*PGS*Physical activity* (moderate vs low)		-0.058 (0.009)	**$1.03e^{-11}$**	0.011 (0.017)	.531	-0.033 (0.011)	**.003**	-0.061 (0.013)	**$4.03e^{-06}$**
	*PGS*Physical activity* (high vs low)		-0.079 (0.009)	**$6.35e^{-18}$**	0.017 (0.018)	.356	-0.047 (0.012)	**$7.31e^{-05}$**	-0.090 (0.014)	**$4.19e^{-10}$**
**5.**	**Education**	110 787								
	BMI PGS	(52 560)	0.239 (0.003)				0.171 (0.004)		0.230 (0.005)	
	College graduate vs non-graduate		-0.241 (0.006)				-0.243 (0.008)		-0.249 (0.010)	
	*PGS*College graduate*		-0.025 (0.006)	**$6.33e^{-05}$**	0.015 (0.013)	.245	-0.006 (0.008)	.474	-0.034 (0.010)	**$3.89e^{-04}$**
**6.**	**Income**	96 627								

i.	BMI PGS	(47 677)	0.276 (0.007)				0.199 (0.010)		0.249 (0.011)	
	Mean household income		-0.075 (0.003)				-0.042 (0.004)		-0.110 (0.004)	
	*PGS*Income*		-0.017 (0.003)	**$1.19e^{-10}$**	0.001 (0.005)	.850	-0.011 (0.003)	**.002**	-0.010 (0.004)	**.010**
		96 627								
ii.	BMI PGS	(47 677)	0.262 (0.006)				0.194 (0.009)		0.235 (0.009)	
	Household income, 5 levels		-0.075 (0.003)				-0.042 (0.004)		-0.110 (0.004)	
	*PGS*Income (£18-£31k vs <£18k)*		-0.025 (0.009)	**.004**	-0.020 (0.018)	.254	-0.027 (0.012)	.023	-0.004 (0.013)	.785
	*PGS*Income (£31-£52k vs <£18k)*		-0.032 (0.009)	**$2.21e^{-04}$**	-0.001 (0.017)	.945	-0.018 (0.012)	.115	-0.017 (0.013)	.209
	*PGS*Income (£52-£100k vs <£18k)*		-0.051 (0.009)	**$4.61e^{-08}$**	-0.009 (0.019)	.651	-0.037 (0.012)	**.002**	-0.023 (0.015)	.116
	*PGS*Income (>£100k vs <£18k)*		-0.076 (0.015)	**$6.76e^{-07}$**	0.015 (0.031)	.637	-0.052 (0.019)	**.006**	-0.058 (0.024)	.017
**7.**	**Deprivation**	111 671								
i.	BMI PGS	(52 994)	0.247 (0.003)				0.181 (0.004)		0.234 (0.005)	
	Townsend deprivation index score		0.026 (0.001)				0.014 (0.001)		0.039 (0.001)	
	*PGS*Deprivation*		0.009 (0.001)	**$7.62e^{-14}$**	-0.002 (0.002)	.207	0.006 (0.001)	**$7.06e^{-07}$**	0.008 (0.001)	**$5.60e^{-08}$**
		111 671								
ii.	BMI PGS	(52 994)	0.210 (0.006)				0.150 (0.008)		0.208 (0.009)	
	Townsend quartiles		0.066 (0.003)				0.039 (0.003)		0.092 (0.004)	
	*PGS*Deprivation (2nd quartile vs 1st)*		0.007 (0.008)	.398	0.005 (0.016)	.750	0.012 (0.011)	.241	-0.004 (0.012)	.747
	*PGS*Deprivation (3rd quartile vs 1st)*		0.025 (0.008)	**.002**	0.004 (0.016)	.821	0.026 (0.011)	**.015**	0.007 (0.012)	.543
	*PGS*Deprivation (4th quartile vs 1st)*		0.066 (0.008)	**$2.36e^{-09}$**	-0.005 (0.016)	.742	0.050 (0.011)	**$2.43e^{-06}$**	0.055 (0.012)	**$9.19e^{-06}$**
**8.**	**Cognitive ability**	35 904								
i.	BMI PGS	(17 199)	0.236 (0.016)				0.145 (0.020)		0.238 (0.024)	
	Verbal-numerical reasoning		-0.046 (0.005)				-0.050 (0.007)		-0.045 (0.008)	
	*PGS*Cognitive ability*		-0.001 (0.002)	.787	0.011 (0.005)	.022	0.004 (0.003)	.196	-0.002 (0.004)	.641
		35 904								
ii.	BMI PGS	(17 199)	0.230 (0.007)				0.163 (0.009)		0.224 (0.010)	
	High vs low score		-0.071 (0.010)				-0.079 (0.013)		-0.070 (0.016)	
	*PGS*Cognitive ability (high vs low)*		0.004 (0.010)	.702	0.026 (0.020)	.208	0.013 (0.013)	.319	0.010 (0.016)	.537
**9.**	**Neuroticism**	107 719								
i.	BMI PGS	(50 797)	0.230 (0.005)				0.175 (0.006)		0.215 (0.008)	
	Neuroticism score		0.008 (0.001)				0.003 (0.001)		0.012 (0.001)	
	*PGS*Neuroticism*		0.002 (0.001)	.058	-0.002 (0.002)	.228	-0.001 (0.001)	.543	0.002 (0.001)	.085
		107 719								
ii.	BMI PGS	(50 797)	0.236 (0.006)				0.177 (0.007)		0.220 (0.008)	
	Neuroticism quartiles		0.022 (0.003)				0.009 (0.003)		0.033 (0.004)	
	*PGS*Neuroticism (2nd quartile vs 1st)*		-0.003 (0.008)	.700	0.014 (0.015)	.371	-0.015 (0.010)	.162	0.003 (0.012)	.813
	*PGS*Neuroticism (3rd quartile vs 1st)*		-0.002 (0.009)	.809	0.019 (0.018)	.289	0.007 (0.010)	.469	-0.002 (0.012)	.873
	*PGS*Neuroticism (4th quartile vs 1st)*		0.010 (0.008)	.209	-0.016 (0.017)	.336	-0.017 (0.011)	.139	0.024 (0.013)	.055

All models include: age, age^2, genetic batch, genetic array, assessment centre, and 10 principal components of population structure (and sex for total sample). Significance levels for BMI PGS in the models are all p <.001. Statistically significant *p*-values for interaction terms (n=104) are indicated in bold font (FDR correction: $p \leq .015$). Model 3i excludes non-drinkers and low drinkers (<1 unit per day) due to non-linearity of association between alcohol consumption and adiposity.

S8 Table

Models predicting WHR from WHR_{adjBMI} PGS, lifestyle and psychosocial factors, and their two-way interactions (in total sample, men, and women), and additionally, three-way interactions with sex (total sample)

Model		Total N (men, n)	Total sample		3-way term with sex (m)		Men		Women	
			**β (SE)**	**P**	**β (SE)**	**P**	**β (SE)**	**P**	**β (SE)**	**P**
**1.**	**Basic model**	111 758								
	WHR_{adjBMI} PGS	(53 033)	0.089 (0.002)				0.056 (0.002)		0.102 (0.003)	
**2.**	**Smoking**	111 464								
	WHR_{adjBMI} PGS	(52 900)	0.089 (0.003)				0.053 (0.003)		0.101 (0.004)	
	Smoking status (never, previous, current)		0.118 (0.003)				0.106 (0.003)		0.129 (0.004)	
	*PGS*Smoking (previous vs never)*		-0.006 (0.004)	.147	0.000 (0.008)	.961	0.002 (0.005)	.668	-0.001 (0.006)	.881
	*PGS*Smoking (current vs never)*		0.008 (0.006)	.170	0.012 (0.012)	.306	0.009 (0.007)	.238	0.000 (0.009)	.994
**3.**	**Alcohol**	61 348								
i.	WHR_{adjBMI} PGS	(34 852)	0.092 (0.005)				0.057 (0.006)		0.095 (0.008)	
	Alcohol intake, gpd		0.004 (0.000)				0.002 (0.000)		0.006 (0.000)	
	*PGS*Alcohol intake*		-0.000 (0.000)	.035	-0.001 (0.000)	.136	-0.000 (0.000)	.722	0.000 (0.000)	.331
		91 002								
ii.	WHR_{adjBMI} PGS	(44 326)	0.090 (0.004)				0.057 (0.006)		0.101 (0.006)	
	Alcohol intake, 5-levels		0.020 (0.001)				0.013 (0.002)		0.027 (0.002)	
	*PGS*Alcohol intake (none vs 8-16g)*		0.015 (0.008)	.069	-0.012 (0.017)	.475	0.002 (0.012)	.893	0.012 (0.011)	.265
	*PGS*Alcohol intake (<8g vs 8-16g)*		0.010 (0.006)	.099	-0.010 (0.012)	.429	-0.002 (0.009)	.835	0.001 (0.008)	.848
	*PGS*Alcohol intake (>24g vs 8-16g)*		-0.009 (0.007)	.174	-0.021 (0.013)	.118	-0.005 (0.008)	.581	0.001 (0.010)	.906
	*PGS*Alcohol intake (16-24g vs 8-16g)*		-0.011 (0.006)	.065	-0.025 (0.012)	.040	-0.001 (0.007)	.857	0.009 (0.010)	.401
**4.**	**Physical activity**	87 296								
i.	WHR_{adjBMI} PGS	(43 518)	0.094 (0.004)				0.063 (0.005)		0.091 (0.007)	
	Physical activity, METS mins/week		-0.001 (0.000)				-0.002 (0.000)		-0.001 (0.000)	
	*PGS*Physical activity*		-0.000 (0.000)	.067	-0.000 (0.000)	.036	-0.000 (0.000)	.082	0.000 (0.000)	.137
		87 296								
ii.	WHR_{adjBMI} PGS	(43 518)	0.095 (0.005)				0.058 (0.006)		0.096 (0.007)	
	Physical activity groups		-0.055 (0.003)				-0.078 (0.004)		-0.032 (0.005)	
	*PGS*Physical activity* (moderate vs low)		-0.006 (0.006)	.320	-0.003 (0.011)	.775	0.002 (0.007)	.786	0.002 (0.009)	.834
	*PGS*Physical activity* (high vs low)		-0.013 (0.006)	.029	-0.020 (0.012)	.097	-0.013 (0.008)	.091	0.008 (0.009)	.422
**5.**	**Education**	110 731								
	WHR_{adjBMI} PGS	(52 530)	0.089 (0.002)				0.055 (0.003)		0.105 (0.003)	
	College graduate vs non-graduate		-0.080 (0.004)				-0.097 (0.005)		-0.062 (0.006)	
	*PGS*College graduate*		-0.004 (0.004)	.353	0.017 (0.008)	.039	0.001 (0.005)	.890	-0.011 (0.006)	.085
**6.**	**Income**	96 584								

i.	WHR_{adjBMI} PGS	(47 651)	0.098 (0.005)				0.057 (0.006)		0.121 (0.007)	
	Mean household income		-0.049 (0.002)				-0.055 (0.002)		-0.047 (0.003)	
	*PGS*Income*		-0.005 (0.002)	.007	0.011 (0.003)	.001	-0.001 (0.002)	.582	-0.009 (0.003)	7.40e^{-04}
		96 584								
ii.	WHR_{adjBMI} PGS		0.099 (0.004)				0.060 (0.006)		0.114 (0.006)	
	Household income, 5 levels		-0.048 (0.002)				-0.055 (0.002)		-0.047 (0.003)	
	*PGS*Income (£18-£31k vs <£18k)*		-0.016 (0.006)	.006	0.006 (0.011)	.629	-0.014 (0.069)	.069	-0.011 (0.008)	.197
	*PGS*Income (£31-£52k vs <£18k)*		-0.018 (0.006)	.002	0.021 (0.011)	.063	-0.002 (0.007)	.769	-0.020 (0.008)	.020
	*PGS*Income (£52-£100k vs <£18k)*		-0.017 (0.006)	.004	0.034 (0.012)	.005	-0.007 (0.008)	.356	-0.029 (0.009)	.002
	*PGS*Income (>£100k vs <£18k)*		-0.015 (0.010)	.132	0.031 (0.020	.124	-0.013 (0.012)	.293	-0.026 (0.016)	.093
**7.**	**Deprivation**	111 615								
i.	WHR_{adjBMI} PGS	(52 964)	0.091 (0.002)				0.058 (0.003)		0.105 (0.003)	
	Townsend deprivation index score		0.016 (0.001)				0.017 (0.001)		0.018 (0.001)	
	*PGS*Deprivation*		0.001 (0.001)	.031	-0.000 (0.001)	.996	0.001 (0.001)	.066	0.002 (0.001)	.033
		111 615								
ii.	WHR_{adjBMI} PGS	(52 964)	0.081 (0.004)				0.054 (0.005)		0.089 (0.006)	
	Townsend quartiles		0.038 (0.002)				0.039 (0.002)		0.041 (0.003)	
	*PGS*Deprivation (2nd quartile vs 1st)*		0.008 (0.005)	.109	-0.004 (0.011)	.698	-0.001 (0.007)	.912	0.010 (0.008)	.188
	*PGS*Deprivation (3rd quartile vs 1st)*		0.011 (0.005)	.030	-0.007 (0.011)	.520	0.001 (0.007)	.844	0.022 (0.008)	.004
	*PGS*Deprivation (4th quartile vs 1st)*		0.013 (0.005)	.013	-0.006 (0.011)	.581	0.007 (0.007)	.286	0.021 (0.008)	.007
**8.**	**Cognitive ability**	35 881								
i.	WHR_{adjBMI} PGS	(17 184)	0.101 (0.010)				0.059 (0.013)		0.096 (0.015)	
	Verbal-numerical reasoning		-0.031 (0.003)				-0.030 (0.004)		-0.031 (0.005)	
	*PGS*Cognitive ability*		-0.002 (0.002)	.285	0.001 (0.003)	.789	0.000 (0.002)	.824	0.001 (0.002)	.596
		35 881								
ii.	WHR_{adjBMI} PGS	(17 184)	0.094 (0.004)				0.060 (0.006)		0.104 (0.007)	
	High vs low score		-0.044 (0.007)				-0.045 (0.009)		-0.042 (0.010)	
	*PGS*Cognitive ability (high vs low)*		-0.004 (0.007)	.526	-0.002 (0.013)	.898	0.004 (0.009)	.681	0.001 (0.010)	.945
**9.**	**Neuroticism**	107 663								
i.	WHR_{adjBMI} PGS	(50 767)	0.082 (0.003)				0.053 (0.004)		0.095 (0.005)	
	Neuroticism score		0.010 (0.001)				0.013 (0.001)		0.009 (0.001)	
	*PGS*Neuroticism*		0.002 (0.001)	.003	0.001 (0.001)	.349	0.001 (0.001)	.345	0.001 (0.001)	.119
		107 663								
ii.	WHR_{adjBMI} PGS	(50 767)	0.085 (0.004)				0.056 (0.004)		0.099 (0.005)	
	Neuroticism quartiles		0.027 (0.002)				0.033 (0.002)		0.023 (0.003)	
	*PGS*Neuroticism (2nd quartile vs 1st)*		0.001 (0.005)	.784	0.002 (0.010)	.874	-0.010 (0.007)	.138	-0.003 (0.008)	.664
	*PGS*Neuroticism (3rd quartile vs 1st)*		-0.001 (0.006)	.863	0.002 (0.012)	.855	-0.002 (0.007)	.809	0.003 (0.007)	.696
	*PGS*Neuroticism (4th quartile vs 1st)*		0.016 (0.005)	.003	0.011 (0.011)	.325	0.008 (0.007)	.270	0.012 (0.008)	.138

All models include: age, age^2, BMI, genetic batch, genetic array, assessment centre, and 10 principal components of population structure (and sex for total sample). Significance levels for WHR_{adjBMI} in the models are all $p<.001$. After FDR correction of p-values for 104 interaction terms there were none that met statistical significance. Model 3i excludes non-drinkers and low drinkers (<1 unit per day) due to non-linearity of association between alcohol consumption and adiposity.

S9 Table

Models predicting WHR from WHR PGS, lifestyle and psychosocial factors, and their two-way interactions (in total sample, men, and women), and additionally, three-way interactions with sex (total sample)

**Model**			**Total N (men, n)**	**Total sample**		**3-way term with sex (m)**		**Men**		**Women**	
				**β (SE)**	**P**	**β (SE)**	**P**	**β (SE)**	**P**	**β (SE)**	**P**
**1.**	**Basic model**		111 758								
		WHR PGS	(53 033)	0.070 (0.002)				0.039 (0.002)		0.083(0.003)	
**2.**	**Smoking**		111 464								
		WHR PGS	(52 900)	0.068 (0.003)				0.037 (0.003)		0.081 (0.004)	
		Smoking status (never, previous, current)		0.117 (0.003)				0.106 (0.003)		0.128 (0.004)	
		*PGS*Smoking (previous vs never)*		-0.002 (0.004)	.566	-0.003 (0.008)	.735	-0.003 (0.005)	.571	-0.002 (0.006)	.785
		*PGS*Smoking (current vs never)*		0.010 (0.006)	.077	0.002 (0.012)	.880	0.010 (0.008)	.176	0.007 (0.009)	.430
**3.**	**Alcohol**		61 348								
i.		WHR PGS	(34 852)	0.069 (0.005)				0.042 (0.006)		0.077 (0.008)	
		Alcohol intake, gpd		0.004 (0.002)				0.002 (0.000)		0.006 (0.000)	
		*PGS*Alcohol intake*		-0.000 (0.000)	.204	-0.001 (0.000)	.018	-0.000 (0.000)	.319	0.000 (0.000)	.279
			91 002								
ii.		WHR PGS	(44 326)	0.069 (0.004)				0.041 (0.006)		0.082 (0.006)	
		Alcohol intake, 5-levels		0.020 (0.001)				0.013 (0.002)		0.027 (0.002)	
		*PGS*Alcohol intake (none vs 8-16g)*		0.017 (0.008)	.038	-0.031 (0.017)	.066	0.000 (0.012)	.993	0.007 (0.011)	.525
		*PGS*Alcohol intake (<8g vs 8-16g)*		0.006 (0.006)	.306	0.001 (0.013)	.956	0.006 (0.009)	.478	-0.003 (0.008)	.720
		*PGS*Alcohol intake (>24g vs 8-16g)*		-0.005 (0.007)	.402	-0.029 (0.013)	.027	-0.006 (0.008)	.472	0.006 (0.010)	.541
		*PGS*Alcohol intake (16-24g vs 8-16g)*		-0.007 (0.006)	.242	-0.032 (0.012)	.010	-0.005 (0.007)	.466	0.010 (0.010)	.332
**4.**	**Physical activity**		87 296								
i.		WHR PGS	(43 518)	0.072 (0.004)				0.045 (0.005)		0.073 (0.007)	
		Physical activity, METS mins/week		-0.001 (0.000)				-0.002 (0.000)		-0.001 (0.000)	
		*PGS*Physical activity*		-0.000 (0.000)	.358	-0.000 (0.000)	.010	-0.000 (0.000)	.151	0.000 (0.000)	.172
			87 296								
ii.		WHR PGS	(43 518)	0.073 (0.005)				0.038 (0.006)		0.078 (0.008)	
		Physical activity groups		-0.057 (0.003)				-0.079 (0.004)		-0.033 (0.005)	
		*PGS*Physical activity* (moderate vs low)		-0.003 (0.006)	.621	0.004 (0.011)	.737	0.007 (0.007)	.332	0.001 (0.009)	.922
		*PGS*Physical activity* (high vs low)		-0.007 (0.006)	.228	-0.023 (0.012)	.061	-0.010 (0.008)	.205	0.008 (0.009)	.424
**5.**	**Education**		110 731								
		WHR PGS	(52 530)	0.069 (0.002)				0.037 (0.003)		0.085 (0.003)	
		College graduate vs non-graduate		-0.081 (0.004)				-0.098 (0.005)		-0.062 (0.006)	
		*PGS*College graduate*		0.000 (0.004)	.944	0.014 (0.008)	.094	0.002 (0.005)	.659	-0.011 (0.006)	.071
**6.**	**Income**		96 584								
i.		WHR PGS	(47 651)	0.071 (0.005)				0.034 (0.006)		0.094 (0.007)	

	Mean household income		-0.049 (0.002)				-0.055 (0.002)		-0.047 (0.003)	
	*PGS*Income*		-0.002 (0.002)	.276	0.006 (0.003)	.056	0.001 (0.002)	.549	-0.006 (0.003)	.022
		96 584								
ii.	WHR PGS	(47 651)	0.075 (0.004)				0.040 (0.006)		0.089 (0.006)	
	Household income, 5 levels		-0.049 (0.002)				-0.055 (0.002)		-0.047 (0.003)	
	*PGS*Income (£18-£31k vs <£18k)*		-0.009 (0.006)	.102	0.008 (0.012)	.493	-0.011 (0.008)	.155	-0.006 (0.008)	.470
	*PGS*Income (£31-£52k vs <£18k)*		-0.014 (0.006)	.012	0.015 (0.011)	.182	-0.002 (0.007)	.752	-0.012 (0.008)	.142
	*PGS*Income (£52-£100k vs <£18k)*		-0.008 (0.006)	.172	-0.020 (0.012)	.100	0.001 (0.008)	.940	-0.020 (0.009)	.036
	*PGS*Income (>£100k vs <£18k)*		-0.000 (0.010)	.986	0.019 (0.020)	.343	0.000 (0.012)	.988	-0.018 (0.015)	.236
**7.**	**Deprivation**	111 615								
i.	WHR PGS	(52 964)	0.072 (0.002)				0.040 (0.003)		0.085 (0.003)	
	Townsend deprivation index score		0.016 (0.001)				0.017 (0.001)		0.018 (0.001)	
	*PGS*Deprivation*		0.001 (0.001)	.033	0.001 (0.001)	.273	0.001 (0.001)	.374	0.001 (0.001)	.132
		111 615								
ii.	WHR PGS	(52 964)	0.061 (0.004)				0.042 (0.005)		0.073 (0.006)	
	Townsend quartiles		0.038 (0.002)				0.040 (0.002)		0.040 (0.003)	
	*PGS*Deprivation (2nd quartile vs 1st)*		0.010 (0.005)	.065	-0.017 (0.011)	.197	-0.007 (0.007)	.279	0.005 (0.008)	.539
	*PGS*Deprivation (3rd quartile vs 1st)*		0.010 (0.005)	.059	-0.007 (0.011)	.523	-0.002 (0.007)	.733	0.019 (0.008)	.017
	*PGS*Deprivation (4th quartile vs 1st)*		0.016 (0.005)	.003	0.001 (0.011)	.897	-0.000 (0.007)	.943	0.017 (0.008)	.031
**8.**	**Cognitive ability**	35 881								
i.	WHR PGS	(17 184)	0.069 (0.010)				0.037 (0.013)		0.066 (0.016)	
	Verbal-numerical reasoning		-0.031 (0.003)				-0.030 (0.004)		-0.031 (0.005)	
	*PGS*Cognitive ability*		0.000 (0.002)	.831	-0.000 (0.003)	.974	0.000 (0.002)	.851	0.002 (0.002)	.386
		35 881								
ii.	WHR PGS	(17 184)	0.070 (0.004)				0.037 (0.006)		0.077 (0.007)	
	High vs low score		-0.043 (0.007)				-0.044 (0.009)		-0.043 (0.010)	
	*PGS*Cognitive ability (high vs low)*		0.002 (0.007)	.780	-0.001 (0.013)	.252	0.006 (0.009)	.498	0.005 (0.010)	.593
**9.**	**Neuroticism**	107 663								
i.	WHR PGS	(50 767)	0.065 (0.003)				0.036 (0.004)		0.078 (0.005)	
	Neuroticism score		0.011 (0.001)				0.013 (0.001)		0.009 (0.001)	
	*PGS*Neuroticism*		0.001 (0.001)	.016	0.001 (0.001)	.274	0.001 (0.001)	.221	0.001 (0.001)	.250
		107 663								
ii.	WHR PGS	(50 767)	0.066 (0.004)				0.040 (0.004)		0.083 (0.005)	
	Neuroticism quartiles		0.028 (0.002)				0.033 (0.002)		0.024 (0.003)	
	*PGS*Neuroticism (2nd quartile vs 1st)*		0.004 (0.005)	.436	-0.004 (0.010)	.715	-0.010 (0.007)	.151	-0.005 (0.008)	.554
	*PGS*Neuroticism (3rd quartile vs 1st)*		-0.004 (0.006)	.501	-0.003 (0.012)	.800	-0.005 (0.007)	.485	-0.000 (0.007)	.995
	*PGS*Neuroticism (4th quartile vs 1st)*		0.017 (0.005)	.002	0.010 (0.011)	.350	0.012 (0.007)	.086	0.006 (0.008)	.454

All models include: age, age^2, BMI, genetic batch, genetic array, assessment centre, and 10 principal components of population structure (and sex for total sample). Significance levels for WHR PGS in the models are all p <.001. After FDR correction of p-values for 104 interaction terms there were none that met statistical significance. Model 3i excludes non-drinkers and low drinkers (<1 unit per day) due to non-linearity of association between alcohol consumption and adiposity.

S10 Table

Models predicting WHR from WHR_{adjBMI} PGS, lifestyle and psychosocial factors, and their two-way interactions (in total sample, men, and women), and additionally, three-way interactions with sex (total sample). BMI is not included as a covariate.

Model		Total N (men, n)	Total sample		3-way term with sex (m)		Men		Women	
			**β (SE)**	**P**	**β (SE)**	**P**	**β (SE)**	**P**	**β (SE)**	**P**
**1.**	**Basic model**	111 951								
	WHR_{adjBMI} PGS	(53 145)	0.089 (0.002)				0.057 (0.003)		0.098 (0.003)	
**2.**	**Smoking**	111 654								
	WHR_{adjBMI} PGS	(53 010)	0.090 (0.003)				0.055 (0.004)		0.098 (0.004)	
	Smoking status (never, previous, current)		0.132 (0.003)				0.125 (0.004)		0.141 (0.005)	
	*PGS*Smoking (previous vs never)*		-0.006 (0.005)	.182	-0.006 (0.010)	.525	0.004 (0.007)	.557	-0.000 (0.007)	.972
	*PGS*Smoking (current vs never)*		-0.009 (0.007)	.180	0.009 (0.014)	.506	-0.010 (0.009)	.267	-0.009 (0.010)	.374
**3.**	**Alcohol**	61 425								
i.	WHR_{adjBMI} PGS	(34 901)	0.092 (0.006)				0.062 (0.007)		0.095 (0.009)	
	Alcohol intake, gpd		0.005 (0.000)				0.004 (0.000)		0.007 (0.000)	
	*PGS*Alcohol intake*		-0.000 (0.000)	.046	-0.001 (0.000)	.094	-0.000 (0.000)	.418	0.000 (0.000)	.562
		91 146								
ii.	WHR_{adjBMI} PGS	(44 409)	0.092 (0.005)				0.063 (0.007)		0.102 (0.006)	
	Alcohol intake, 5-levels		0.025 (0.002)				0.024 (0.002)		0.027 (0.003)	
	*PGS*Alcohol intake (none vs 8-16g)*		0.018 (0.001)	.062	-0.020 (0.020)	.313	-0.001 (0.015)	.968	0.006 (0.012)	.603
	*PGS*Alcohol intake (<8g vs 8-16g)*		0.006 (0.007)	.361	-0.005 (0.014)	.738	-0.006 (0.011)	.568	-0.009 (0.009)	.324
	*PGS*Alcohol intake (>24g vs 8-16g)*		-0.014 (0.008)	.076	-0.015 (0.015)	.316	-0.010 (0.011)	.365	-0.008 (0.011)	.494
	*PGS*Alcohol intake (16-24g vs 8-16g)*		0.012 (0.007)	.066	-0.031 (0.014)	.032	-0.008 (0.009)	.386	0.006 (0.011)	.610
**4.**	**Physical activity**	87 427								
i.	WHR_{adjBMI} PGS	(43 594)	0.096 (0.005)				0.068 (0.007)		0.086 (0.008)	
	Physical activity, METS mins/week		-0.003 (0.000)				-0.003 (0.000)		-0.003 (0.000)	
	*PGS*Physical activity*		-0.000 (0.000)	.038	-0.000 (0.000)	.045	-0.000 (0.000)	.046	0.000 (0.000)	.158
		87 427								
ii.	WHR_{adjBMI} PGS	(43 594)	0.094 (0.006)				0.061 (0.008)		0.091 (0.008)	
	Physical activity groups		-0.125 (0.003)				-0.144 (0.005)		-0.105 (0.005)	
	*PGS*Physical activity* (moderate vs low)		-0.006 (0.007)	.362	-0.019 (0.013)	.149	0.001 (0.009)	.933	0.003 (0.010)	.732
	*PGS*Physical activity* (high vs low)		-0.042 (0.007)	.045	-0.026 (0.014)	.070	-0.017 (0.010)	.076	0.011 (0.011)	.312
**5.**	**Education**	110 916								
	WHR_{adjBMI} PGS	(52 636)	0.086 (0.003)				0.052 (0.004)		0.099 (0.004)	
	College graduate vs non-graduate		-0.180 (0.005)				-0.219 (0.007)		-0.148 (0.007)	
	*PGS*College graduate*		-0.001 (0.005)	.815	0.021 (0.009)	.026	0.007 (0.007)	.297	-0.009 (0.007)	.180
**6.**	**Income**	96 747								
i.	WHR_{adjBMI} PGS	(47 749)	0.087 (0.006)				0.044 (0.008)		0.108 (0.008)	

	Mean household income		-0.080 (0.002)				-0.076 (0.003)		-0.085 (0.003)	
	PGS*Income		-0.001 (0.002)	.662	0.013 (0.004)	**.001**	0.004 (0.003)	.172	-0.006 (0.003)	.049
		96 747								
ii.	WHR$_{adjBMI}$ PGS	(47 749)	0.089 (0.005)				0.051 (0.007)		0.101 (0.007)	
	Household income, 5 levels		-0.080 (0.002)				-0.076 (0.003)		-0.085 (0.003)	
	PGS*Income (£18-£31k vs <£18k)		-0.007 (0.007)	.300	0.007 (0.013)	.582	-0.007 (0.008)	.439	-0.002 (0.009)	.833
	PGS*Income (£31-£52k vs <£18k)		-0.006 (0.007)	.361	0.024 (0.013)	.066	-0.011 (0.009)	.248	-0.007 (0.009)	.486
	PGS*Income (£52-£100k vs <£18k)		-0.004 (0.007)	.543	0.048 (0.014)	**.001**	0.011 (0.010)	.270	-0.021 (0.010)	.045
	PGS*Income (>£100k vs <£18k)		-0.003 (0.012)	.812	0.029 (0.023)	.218	-0.001 (0.015)	.933	-0.014 (0.018)	.418
**7.**	**Deprivation**	111 807								
i.	WHR$_{adjBMI}$ PGS	(53 075)	0.090 (0.002)				0.058 (0.003)		0.101 (0.004)	
	Townsend deprivation index score		0.027 (0.001)				0.024 (0.001)		0.031 (0.001)	
	PGS*Deprivation		0.001 (0.001)	.117	0.000 (0.001)	.835	0.001 (0.001)	.255	0.002 (0.001)	.061
		111 807								
ii.	WHR$_{adjBMI}$ PGS	(53 075)	0.081 (0.004)				0.050 (0.006)		0.085 (0.006)	
	Townsend quartiles		0.065 (0.002)				0.060 (0.003)		0.072 (0.003)	
	PGS*Deprivation (2nd quartile vs 1st)		0.008 (0.006)	.218	0.002 (0.012)	.845	0.008 (0.009)	.363	0.013 (0.009)	.147
	PGS*Deprivation (3rd quartile vs 1st)		0.008 (0.006)	.190	0.004 (0.012)	.750	0.011 (0.009)	.194	0.020 (0.009)	.021
	PGS*Deprivation (4th quartile vs 1st)		0.012 (0.006)	.056	-0.000 (0.012)	.989	0.010 (0.009)	.264	0.021 (0.009)	.020
**8.**	**Cognitive ability**	35 950								
i.	WHR$_{adjBMI}$ PGS	(17 228)	0.103 (0.012)				0.055 (0.016)		0.097 (0.017)	
	Verbal-numerical reasoning		-0.052 (0.004)				-0.057 (0.005)		-0.048 (0.006)	
	PGS*Cognitive ability		-0.002 (0.002)	.317	0.004 (0.004)	.243	0.001 (0.002)	.705	0.000 (0.003)	.905
		35 950								
ii.	WHR$_{adjBMI}$ PGS	(17 228)	0.094 (0.005)				0.058 (0.007)		0.100 (0.007)	
	High vs low score		-0.076 (0.008)				-0.086 (0.011)		-0.069 (0.010)	
	PGS*Cognitive ability (high vs low)		-0.005 (0.008)	.504	0.011 (0.016)	.481	0.006 (0.011)	.559	-0.003 (0.011)	.773
**9.**	**Neuroticism**	107 845								
i.	WHR$_{adjBMI}$ PGS	(50 875)	0.078 (0.004)				0.048 (0.005)		0.089 (0.005)	
	Neuroticism score		0.014 (0.001)				0.014 (0.001)		0.013 (0.001)	
	PGS*Neuroticism		0.003 (0.001)	**$2.91e^{-04}$**	0.000 (0.001)	.766	0.002 (0.001)	.026	0.002 (0.001)	.053
		107 845								
ii.	WHR$_{adjBMI}$ PGS	(50 875)	0.083 (0.004)				0.053 (0.005)		0.094 (0.006)	
	Neuroticism quartiles		0.035 (0.002)				0.037 (0.003)		0.034 (0.003)	
	PGS*Neuroticism (2nd quartile vs 1st)		-0.002 (0.006)	.771	0.006 (0.012)	.599	-0.005 (0.008)	.547	-0.004 (0.009)	.661
	PGS*Neuroticism (3rd quartile vs 1st)		0.003 (0.007)	.637	0.008 (0.014)	.592	0.003 (0.008)	.759	0.003 (0.008)	.678
	PGS*Neuroticism (4th quartile vs 1st)		0.020 (0.006)	.002	0.004 (0.013)	.736	0.019 (0.009)	.034	0.017 (0.009)	.065

All models include: age, age^2, genetic batch, genetic array, assessment centre, and 10 principal components of population structure (and sex for total sample). Significance levels for WHR$_{adjBMI}$ PGS in the models are all p <.001. Statistically significant p-values for interaction terms (n=104) are indicated in bold font (FDR correction: $p \leq .001$). Model 3i excludes non-drinkers and low drinkers (<1 unit per day) due to non-linearity of association between alcohol consumption and adiposity.

S11 Table

Models predicting WHR from WHR PGS, lifestyle and psychosocial factors, and their two-way interactions (in total sample, men, and women), and additionally, three-way interactions with sex (total sample). BMI is not included as a covariate.

Model	Total N (men, n)	Total sample		3-way term with sex (m)		Men		Women	
		β (SE)	P	*β* (SE)	P	*β* (SE)	P	*β* (SE)	P
1. **Basic model**	111 951								
WHR PGS	(53 145)	0.109 (0.002)				0.081 (0.003)		0.107 (0.003)	
2. **Smoking**	111 654								
WHR PGS	(53 010)	0.107 (0.003)				0.080 (0.004)		0.104 (0.004)	
Smoking status (never, previous, current)		0.130 (0.003)				0.123 (0.004)		0.139 (0.005)	
*PGS*Smoking (previous vs never)*		-0.002 (0.005)	.666	-0.011 (0.009)	.259	-0.004 (0.007)	.549	0.002 (0.007)	.743
*PGS*Smoking (current vs never)*		-0.006 (0.007)	.420	0.002 (0.014)	.883	0.001 (0.009)	.909	0.000 (0.010)	.996
3. **Alcohol**	61 425								
i. WHR PGS	(34 901)	0.108 (0.006)				0.082 (0.007)		0.099 (0.009)	
Alcohol intake, gpd		0.005 (0.000)				0.004 (0.000)		0.007 (0.000)	
*PGS*Alcohol intake*		-0.000 (0.000)	.052	-0.001 (0.001)	.046	-0.000 (0.000)	.284	0.000 (0.000)	.474
	91 146								
ii. WHR PGS	(44 409)	0.107 (0.005)				0.083 (0.007)		0.104 (0.007)	
Alcohol intake, 5-levels		0.025 (0.002)				0.024 (0.002)		0.027 (0.003)	
*PGS*Alcohol intake (none vs 8-16g)*		0.030 (0.009)	**.002**	-0.054 (0.020)	.006	0.011 (0.015)	.456	0.018 (0.012)	.147
*PGS*Alcohol intake (<8g vs 8-16g)*		0.008 (0.007)	.235	0.002 (0.014)	.865	0.006 (0.011)	.561	-0.005 (0.009)	.534
*PGS*Alcohol intake(>24g vs 8-16g)*		-0.011 (0.008)	.147	-0.026 (0.015)	.089	-0.011 (0.011)	.300	0.001 (0.011)	.934
*PGS*Alcohol intake (16-24g vs 8-16g)*		-0.012 (0.007)	.070	-0.032 (0.014)	.026	-0.010 (0.009)	.266	0.009 (0.011)	.455
4. **Physical activity**	87 427								
i. WHR PGS	(43 594)	0.119 (0.005)				0.095 (0.007)		0.102 (0.008)	
Physical activity, METS mins/week		-0.003 (0.000)				-0.003 (0.000)		-0.003 (0.000)	
*PGS*Physical activity*		-0.000 (0.000)	.006	-0.000 (0.000)	.035	-0.000 (0.000)	.008	0.000 (0.000)	.716
	87 427								
ii. WHR PGS	(43 594)	0.117 (0.006)				0.088 (0.008)		0.107 (0.008)	
Physical activity groups		-0.125 (0.003)				-0.144 (0.005)		-0.105 (0.005)	
*PGS*Physical activity* (moderate vs low)		-0.008 (0.007)	.202	-0.007 (0.013)	.621	-0.000 (0.009)	.990	-0.004 (0.010)	.647
*PGS*Physical activity* (high vs low)		-0.019 (0.007)	.008	-0.026 (0.014)	.070	-0.024 (0.010)	.011	-0.000 (0.011)	.972
5. **Education**	110 916								
WHR PGS	(52 636)	0.105 (0.003)				0.075 (0.004)		0.109 (0.004)	
College graduate vs non-graduate		-0.175 (0.005)				-0.215 (0.007)		-0.145 (0.007)	
*PGS*College graduate*		-0.001 (0.005)	.830	0.022 (0.009)	.023	0.006 (0.007)	.349	-0.015 (0.007)	.030
6. **Income**	96 747								
i. WHR PGS	(47 749)	0.107 (0.006)				0.073 (0.008)		0.115 (0.008)	

	Mean household income		-0.078 (0.002)				-0.076 (0.003)		-0.084 (0.003)	
	*PGS*Income*		-0.001 (0.002)	.482	0.008 (0.004)	.051	0.002 (0.003)	.451	-0.006 (0.003)	.055
		96 747								
ii.	WHR PGS	(47 749)	0.108 (0.005)				0.083 (0.007)		0.106 (0.007)	
	Household income, 5 levels		-0.078 (0.002)				-0.076 (0.003)		-0.084 (0.003)	
	*PGS*Income (£18-£31k vs <£18k)*		-0.005 (0.007)	.486	0.003 (0.013)	.826	-0.016 (0.010)	.097	0.001 (0.009)	.949
	*PGS*Income (£31-£52k vs <£18k)*		-0.010 (0.007)	.144	0.013 (0.013)	.311	-0.006 (0.009)	.545	-0.005 (0.009)	.626
	*PGS*Income (£52-£100k vs <£18k)*		-0.005 (0.007)	.449	0.030 (0.014)	.036	0.004 (0.010)	.648	-0.019 (0.010)	.075
	*PGS*Income (>£100k vs <£18k)*		-0.000 (0.011)	.984	0.011 (0.023)	.645	-0.005 (0.015)	.756	-0.018 (0.017)	.309
**7.**	**Deprivation**	111 807								
i.	WHR PGS	(53 075)	0.111 (0.002)				0.083 (0.003)		0.109 (0.004)	
	Townsend deprivation index score		0.027 (0.001)				0.024 (0.001)		0.030 (0.001)	
	*PGS*Deprivation*		0.003 (0.001)	**3.48e^{-04}**	0.001 (0.001)	.424	0.002 (0.001)	.015	0.002 (0.001)	.047
		111 807								
ii.	WHR PGS	(53 075)	0.096 (0.004)				0.072 (0.006)		0.095 (0.006)	
	Townsend quartiles		0.064 (0.002)				0.059 (0.003)		0.071 (0.003)	
	*PGS*Deprivation (2nd quartile vs 1st)*		0.012 (0.006)	.054	-0.009 (0.012)	.487	0.003 (0.009)	.715	0.006 (0.009)	.513
	*PGS*Deprivation (3rd quartile vs 1st)*		0.010 (0.006)	.121	0.003 (0.012)	.791	0.010 (0.009)	.225	0.017 (0.009)	.050
	*PGS*Deprivation (4th quartile vs 1st)*		0.026 (0.006)	**2.63e^{-05}**	0.004 (0.012)	.726	0.017 (0.009)	.042	0.021 (0.009)	.016
**8.**	**Cognitive ability**	35 950								
i.	WHR PGS	(17 228)	0.111 (0.012)				0.072 (0.016)		0.097 (0.017)	
	Verbal-numerical reasoning		-0.050 (0.004)				-0.055 (0.005)		-0.047 (0.006)	
	*PGS*Cognitive ability*		-0.000 (0.002)	.883	0.004 (0.004)	.288	0.001 (0.002)	.607	0.001 (0.003)	.712
		35 950								
ii.	WHR PGS	(17 228)	0.110 (0.005)				0.075 (0.007)		0.102 (0.007)	
	High vs low score		-0.072 (0.008)				-0.083 (0.012)		-0.068 (0.011)	
	*PGS*Cognitive ability (high vs low)*		0.001 (0.008)	.917	-0.011 (0.016)	.481	0.011 (0.011)	.323	0.003 (0.011)	.782
**9.**	**Neuroticism**	107 845								
i.	WHR PGS	(50 875)	0.100 (0.004)				0.073 (0.005)		0.098 (0.005)	
	Neuroticism score		0.014 (0.001)				0.014 (0.001)		0.013 (0.001)	
	*PGS*Neuroticism*		0.002 (0.001)	**.002**	0.001 (0.001)	.615	0.002 (0.001)	.042	0.002 (0.001)	.051
		107 845								
ii.	WHR PGS	(50 875)	0.105 (0.004)				0.078 (0.005)		0.105 (0.006)	
	Neuroticism quartiles		0.035 (0.002)				0.037 (0.003)		0.034 (0.003)	
	*PGS*Neuroticism (2nd quartile vs 1st)*		-0.001 (0.006)	.875	0.004 (0.012)	.759	-0.010 (0.008)	.239	-0.006 (0.009)	.525
	*PGS*Neuroticism (3rd quartile vs 1st)*		-0.001 (0.007)	.842	0.012 (0.014)	.404	0.007 (0.008)	.429	0.001 (0.008)	.860
	*PGS*Neuroticism (4th quartile vs 1st)*		0.019 (0.006)	**.002**	0.004 (0.013)	.778	0.015 (0.009)	.091	0.015 (0.009)	.108

All models include: age, age^2, genetic batch, genetic array, assessment centre, and 10 principal components of population structure (and sex for total sample). Significance levels for WHR in the models are all $p<.001$. Statistically significant p-values for interaction terms (n=104) are indicated in bold font (FDR correction: $p \leq .002$). Model 3i excludes non-drinkers and low drinkers (<1 unit per day) due to non-linearity of association between alcohol consumption and adiposity.

S12 Table Associations between BMI phenotype and BMI PGS, stratified by lifestyle and psychosocial factors

Moderator	Model terms	Total Sample		Men		Women	
		n	**β (CI), *SE***	*n*	**β (CI), *SE***	*n*	**β (CI), *SE***
Smoking							
	Never	*59 731*	0.234 (0.226, 0.241), 0.004	*26 101*	0.164 (0.154, 0.174), 0.005	*33 630*	0.217 (0.206, 0.229), 0.006
	Previous	*38 214*	0.234 (0.225, 0.244), 0.005	*19 824*	0.176 (0.164, 0.188), 0.006	*18 390*	0.233 (0.217, 0.248), 0.008
	Current	*13 575*	0.242 (0.225, 0.258), 0.008	*7005*	0.178 (0.156, 0.199), 0.011	*6570*	0.231 (0.205, 0.256), 0.013
Alcohol							
	None	*7526*	0.289 (0.263, 0.315), 0.013	*2706*	0.193 (0.153, 0.233), 0.020	*4820*	0.278 (0.243, 0.313), 0.018
	<8g	*22 157*	0.246 (0.233, 0.259), 0.007	*6780*	0.179 (0.157, 0.200), 0.011	*15 377*	0.224 (0.208, 0.241), 0.008
	8-16g	*22 538*	0.211 (0.199, 0.223), 0.006	*9100*	0.160 (0.143, 0.177), 0.009	*13 438*	0.188 (0.172, 0.203), 0.008
	16-24g	*14 691*	0.206 (0.192, 0.220), 0.007	*7903*	0.161 (0.143, 0.179), 0.009	*6788*	0.182 (0.160, 0.204), 0.011
	>24g	*24 133*	0.198 (0.187, 0.208), 0.005	*17 861*	0.165 (0.153, 0.177), 0.006	*6272*	0.193 (0.169, 0.216), 0.012
Physical activity							
	Low	*15 850*	0.282 (0.265, 0.299), 0.009	*7957*	0.203 (0.182, 0.225), 0.011	*7893*	0.274 (0.248, 0.301), 0.014
	Moderate	*43 035*	0.225 (0.216, 0.234), 0.004	*20 682*	0.170 (0.158, 0.182), 0.006	*22 353*	0.216 (0.203, 0.229), 0.007
	High	*28 452*	0.204 (0.194, 0.214), 0.005	*14 900*	0.154 (0.142, 0.167), 0.007	*13 552*	0.189 (0.173, 0.205), 0.008
Education							
	College graduate	*33 762*	0.214 (0.205, 0.224), 0.005	*16 694*	0.165 (0.153, 0.177), 0.006	*17 068*	0.196 (0.181, 0.211), 0.008
	Non-college graduate	*77 025*	0.239 (0.232, 0.246), 0.004	*35 866*	0.170 (0.161, 0.180), 0.005	*41 159*	0.230 (0.220, 0.241), 0.005
Income							
	<£18k	*22 027*	0.264 (0.250, 0.278), 0.007	*9760*	0.195 (0.176, 0.214), 0.010	*12 267*	0.236 (0.216, 0.256), 0.010
	£18-£31k	*25 009*	0.236 (0.224, 0.248), 0.006	*11 938*	0.166 (0.150, 0.182), 0.008	*13 071*	0.231 (0.213, 0.249), 0.009
	£31-£52k	*25 557*	0.230 (0.218, 0.241), 0.006	*12 997*	0.175 (0.161, 0.190), 0.007	*12 560*	0.220 (0.202, 0.238), 0.009
	£52-£100k	*19 209*	0.211 (0.198, 0.223), 0.006	*10 329*	0.156 (0.141, 0.172), 0.008	*8880*	0.213 (0.192, 0.233), 0.010
	>£100k	*4825*	0.179 (0.156, 0.203), 0.012	*2653*	0.140 (0.111, 0.169), 0.015	*2172*	0.170 (0.132, 0.208), 0.019
Deprivation							
	1st quartile (least)	*27 950*	0.210 (0.200, 0.220), 0.005	*13 387*	0.150 (0.136, 0.163), 0.007	*14 563*	0.208 (0.192, 0.224), 0.008
	2nd quartile	*27 946*	0.216 (0.206, 0.227), 0.005	*13 182*	0.162 (0.148, 0.177), 0.007	*14 764*	0.204 (0.188, 0.220), 0.008
	3rd quartile	*27 910*	0.234 (0.223, 0.246), 0.006	*13 030*	0.176 (0.161, 0.191), 0.008	*14 880*	0.215 (0.198, 0.232), 0.009
	4th quartile	*27 865*	0.275 (0.263, 0.288), 0.006	*13 395*	0.200 (0.183, 0.217), 0.009	*14 470*	0.261 (0.242, 0.280), 0.010
Cognitive ability							
	High scores	*15 631*	0.234 (0.219, 0.248), 0.008	*7831*	0.177 (0.158, 0.196), 0.010	*7799*	0.232 (0.209, 0.255), 0.012
	Low scores	*20 274*	0.231 (0.217, 0.244), 0.007	*9368*	0.163 (0.145, 0.181), 0.009	*10 906*	0.225 (0.205, 0.245), 0.010
Neuroticism							
	1st quartile (least)	*29 232*	0.236 (0.225, 0.246), 0.005	*17 098*	0.177 (0.164, 0.190), 0.007	*18 422*	0.221 (0.206, 0.235), 0.008
	2nd quartile	*35 533*	0.232 (0.222, 0.242), 0.005	*11 775*	0.163 (0.147, 0.178), 0.008	*12 598*	0.223 (0.205, 0.241), 0.009
	3rd quartile	*18 332*	0.233 (0.219, 0.247), 0.007	*12 449*	0.185 (0.169, 0.200), 0.008	*15 116*	0.218 (0.201, 0.235), 0.009
	4th quartile	*24 622*	0.247 (0.234, 0.260), 0.007	*9475*	0.161 (0.143, 0.180), 0.009	*10 786*	0.244 (0.223, 0.265), 0.011

All models include: age, age^2, genetic batch, genetic array, assessment centre, and 10 principal components of population structure (and sex for total sample)

S13 Table Associations between WHR phenotype and WHR_{adjBMI} PGS, stratified by lifestyle and psychosocial factors

**Moderator**	**Model terms**	**Total Sample** *n*	**Total Sample** ***β* (CI), *SE***	**Men** *n*	**Men** ***β* (CI), *SE***	**Women** *n*	**Women** ***β* (CI), *SE***
Smoking							
	Never	*59 701*	0.090 (0.085, 0.095), 0.003	*26 088*	0.053 (0.047, 0.060), 0.003	*33 613*	0.102 (0.095, 0.109), 0.004
	Previous	*38 196*	0.083 (0.076, 0.089), 0.003	*19 811*	0.055 (0.047, 0.062), 0.004	*18 385*	0.101 (0.091, 0.110), 0.005
	Current	*13 567*	0.094 (0.083, 0.105), 0.006	*7001*	0.059 (0.046, 0.073), 0.007	*6566*	0.097 (0.080, 0.114), 0.009
Alcohol							
	None	*7514*	0.106 (0.090, 0.121), 0.008	*2701*	0.057 (0.034, 0.080), 0.012	*4813*	0.116 (0.095, 0.136), 0.010
	<8g	*22 140*	0.099 (0.090, 0.107), 0.004	*6773*	0.054 (0.041, 0.068), 0.007	*15 360*	0.102 (0.091, 0.112), 0.005
	8-16g	*22 535*	0.089 (0.081, 0.098), 0.004	*9098*	0.056 (0.045, 0.067), 0.006	*13 437*	0.100 (0.089, 0.111), 0.006
	16-24g	*14 687*	0.082 (0.072, 0.092), 0.005	*7899*	0.052 (0.040, 0.064), 0.006	*6788*	0.102 (0.086, 0.118), 0.008
	>24g	*24 126*	0.079 (0.072, 0.087), 0.004	*17 855*	0.055 (0.047, 0.063), 0.004	*6271*	0.108 (0.091, 0.125), 0.009
Physical activity							
	Low	*15 833*	0.094 (0.084, 0.105), 0.005	*7950*	0.059 (0.046, 0.072), 0.007	*7883*	0.095 (0.079, 0.110), 0.008
	Moderate	*43 015*	0.090 (0.084, 0.096), 0.003	*20 670*	0.060 (0.053, 0.068), 0.004	*22 345*	0.100 (0.091, 0.109), 0.005
	High	*28 448*	0.080 (0.073, 0.087), 0.004	*14 898*	0.046 (0.037, 0.055), 0.004	*13 550*	0.102 (0.091, 0.113), 0.006
Education							
	College graduate	*76 980*	0.089 (0.084, 0.093), 0.002	*35 841*	0.055 (0.049, 0.060), 0.003	*41 139*	0.103 (0.097, 0.110), 0.003
	Non-college graduate	*33 751*	0.087 (0.081, 0.094), 0.003	*16 689*	0.056 (0.048, 0.064), 0.004	*17 062*	0.096 (0.086, 0.106), 0.005
Income							
	<£18k	*22 008*	0.096 (0.087, 0.105), 0.005	*9748*	0.059 (0.047, 0.070), 0.006	*12 260*	0.111 (0.098, 0.123), 0.006
	£18-£31k	*25 000*	0.082 (0.074, 0.090), 0.004	*11 930*	0.046 (0.036, 0.056), 0.005	*13 070*	0.102 (0.090, 0.114), 0.006
	£31-£52k	*25 548*	0.082 (0.074, 0.089), 0.004	*12 994*	0.058 (0.049, 0.067), 0.005	*12 554*	0.096 (0.084, 0.107), 0.006
	£52-£100k	*19 206*	0.083 (0.075, 0.092), 0.004	*10 327*	0.054 (0.044, 0.064), 0.005	*8879*	0.086 (0.073, 0.100), 0.007
	>£100k	*4822*	0.086 (0.069, 0.102), 0.008	*2652*	0.048 (0.028, 0.068), 0.010	*2170*	0.090 (0.062, 0.117), 0.014
Deprivation							
	1st quartile (least)	*27 938*	0.082 (0.075, 0.089), 0.004	*13 378*	0.056 (0.047, 0.065), 0.005	*14 560*	0.091 (0.080, 0.101), 0.005
	2nd quartile	*27 936*	0.091 (0.084, 0.098), 0.004	*13 176*	0.054 (0.045, 0.064), 0.005	*14 760*	0.100 (0.089, 0.111), 0.006
	3rd quartile	*27 894*	0.093 (0.085, 0.100), 0.004	*13 023*	0.056 (0.046, 0.065), 0.005	*14 871*	0.111 (0.100, 0.122), 0.006
	4th quartile	*27 847*	0.090 (0.082, 0.098), 0.004	*13 387*	0.059 (0.049, 0.069), 0.005	*14 460*	0.107 (0.095, 0.118), 0.006
Cognitive ability							
	High scores	*15 620*	0.089 (0.080, 0.099), 0.005	*7825*	0.063 (0.051, 0.075), 0.006	*7795*	0.105 (0.090, 0.120), 0.008
	Low scores	*20 261*	0.093 (0.084, 0.102), 0.005	*9359*	0.060 (0.048, 0.072), 0.006	*10 902*	0.103 (0.090, 0.116), 0.007
Neuroticism							
	1st quartile (least)	*29 215*	0.086 (0.078, 0.093), 0.004	*17 086*	0.056 (0.048, 0.065), 0.004	*18 414*	0.099 (0.090, 0.109), 0.005
	2nd quartile	*35 524*	0.087 (0.081, 0.094), 0.003	*11 772*	0.047 (0.037, 0.057), 0.005	*12 596*	0.098 (0.086, 0.110), 0.006
	3rd quartile	*18 319*	0.083 (0.074, 0.092), 0.005	*12 442*	0.055 (0.045, 0.065), 0.005	*15 108*	0.101 (0.090, 0.112), 0.006
	4th quartile	*24 605*	0.099 (0.091, 0.107), 0.004	*9467*	0.064 (0.052, 0.075), 0.006	*10 778*	0.108 (0.095, 0.121), 0.007

All models include: age, age^2, BMI, genetic batch, genetic array, assessment centre, and 10 principal components of population structure (and sex for total sample)

S14 Table

Descriptive data on lifestyle and psychosocial factors within age groups, mean (SD) or % for categorical variables

		40 to 49 years	50 to 59 years	60 to 73 years
BMI		N=23 973	N=37 536	N=50 310
		27.10 (4.97)	27.63 (4.98)	27.65 (4.57)
WHR		N=24 007	N=37 574	N=50 375
		0.85 (0.09)	0.87 (0.09)	0.89 (0.09)
Smoking status		N=24 027	N=37 534	N=50 291
	never	58.6%	55.1%	50.0%
	previous	24.8%	31.8%	40.6%
	current	16.6%	13.1%	9.4%
Alcohol		N=17 960	N=28 339	N=37 437
	average grams / day	19.17 (15.30)	19.32 (15.53)	*18.60 (15.40)
Physical activity		N=19 892	N=29 640	N=38 052
	average MET mins/week	3160 (4327)	*2948 (4052)	3129 (3682)
College		N=23 918	N=37 372	N=49 824
	graduate	36.4%	34.0%	25.0%
Income		N=22 089	N=33 365	N=41 446
	<£18,000	12.2%	15.7%	34.3%
	£18,000-£30,999	18.4%	22.1%	32.9%
	£31,000-£51,999	31.2%	30.0%	21.0%
	£52,999-£100,000	30.0%	25.8%	9.7%
	>£100,000	8.2%	6.5%	2.1%
Deprivation score		N=24 020	N=37 575	N=50 410
	average	-1.16 (3.11)	-1.47 (2.99)	-1.66 (2.92)
Verbal-numerical reasoning		N=7365	N=11 671	N=16 999
	average (0 to 13)	6.29 (2.10)	6.35 (2.14)	5.97 (2.07)
Neuroticism		N=23 426	N=36 479	N=48 133
	average (0 to 12)	4.44 (3.26)	4.22 (3.26)	3.66 (3.05)

Statistically significant sex differences exist across all variables in the table (p<.01) based upon t-tests for continuous variables and ANOVA or Pearson's chi-square for categorical variables. Asterisks indicate that in post-hoc analyses one group was significantly different from the reference group but not another, i.e. 60-73 year olds but not 50-59 year olds, drank significantly less alcohol than 40-49 year olds. Another non-linear association between age and the moderating variables was for verbal reasoning score, which was significantly higher in the 50-59 years old (vs 40-49 year olds), and significantly lower in the 60-73 year olds.

S1a Fig

Associations between alcohol consumption and adiposity measures allowing for cubic effects (regression curves allowing for restricted cubic splines): (a) BMI in women (top left); (b) BMI in men (top right); (c) WHR in women (bottom left); (d) WHR in men (bottom right).

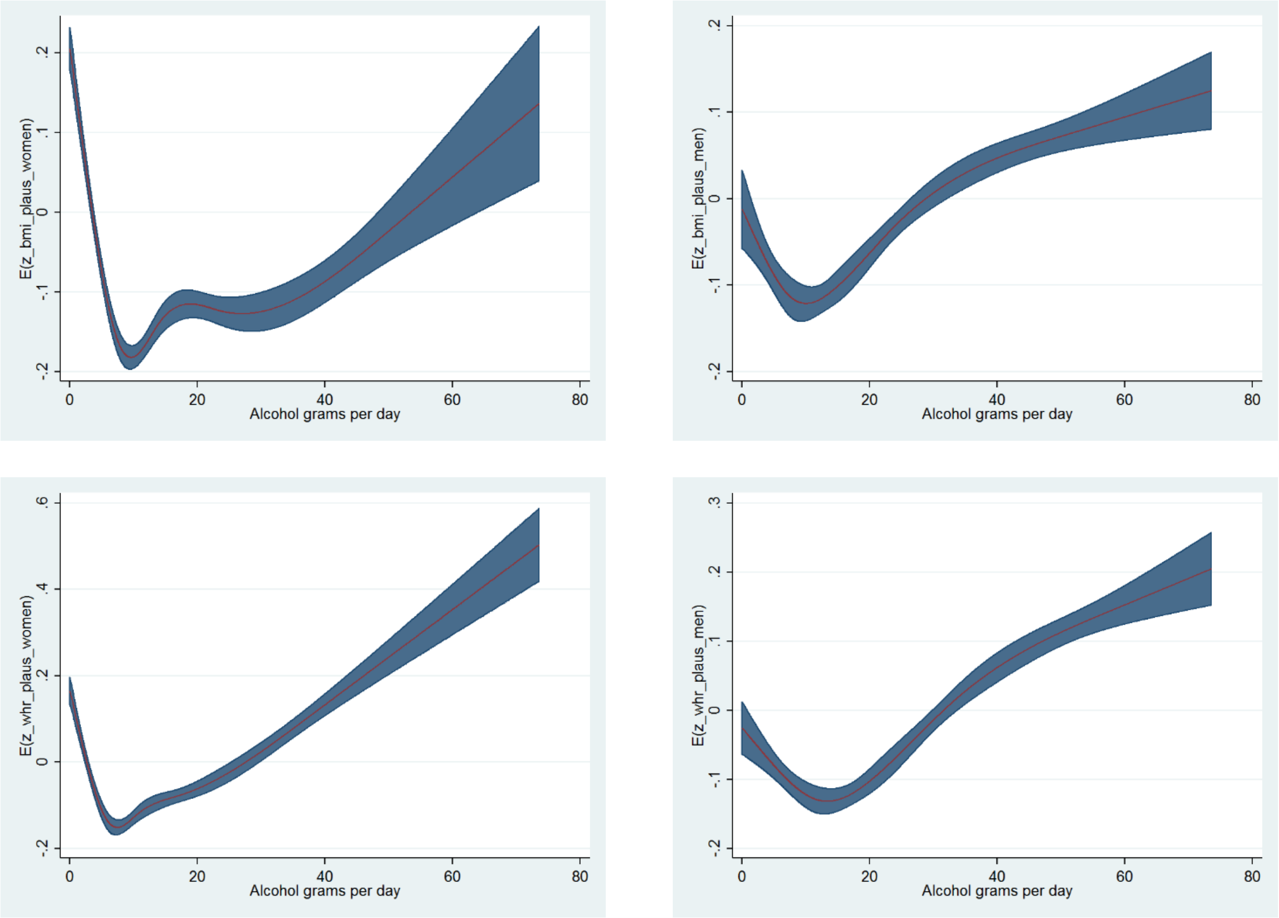

S1b Fig

Associations between physical activity and adiposity measures allowing for cubic effects (regression curves allowing for restricted cubic splines): (a) BMI in women (top left); (b) BMI in men (top right); (c) WHR in women (bottom left); (d) WHR in men (bottom right).

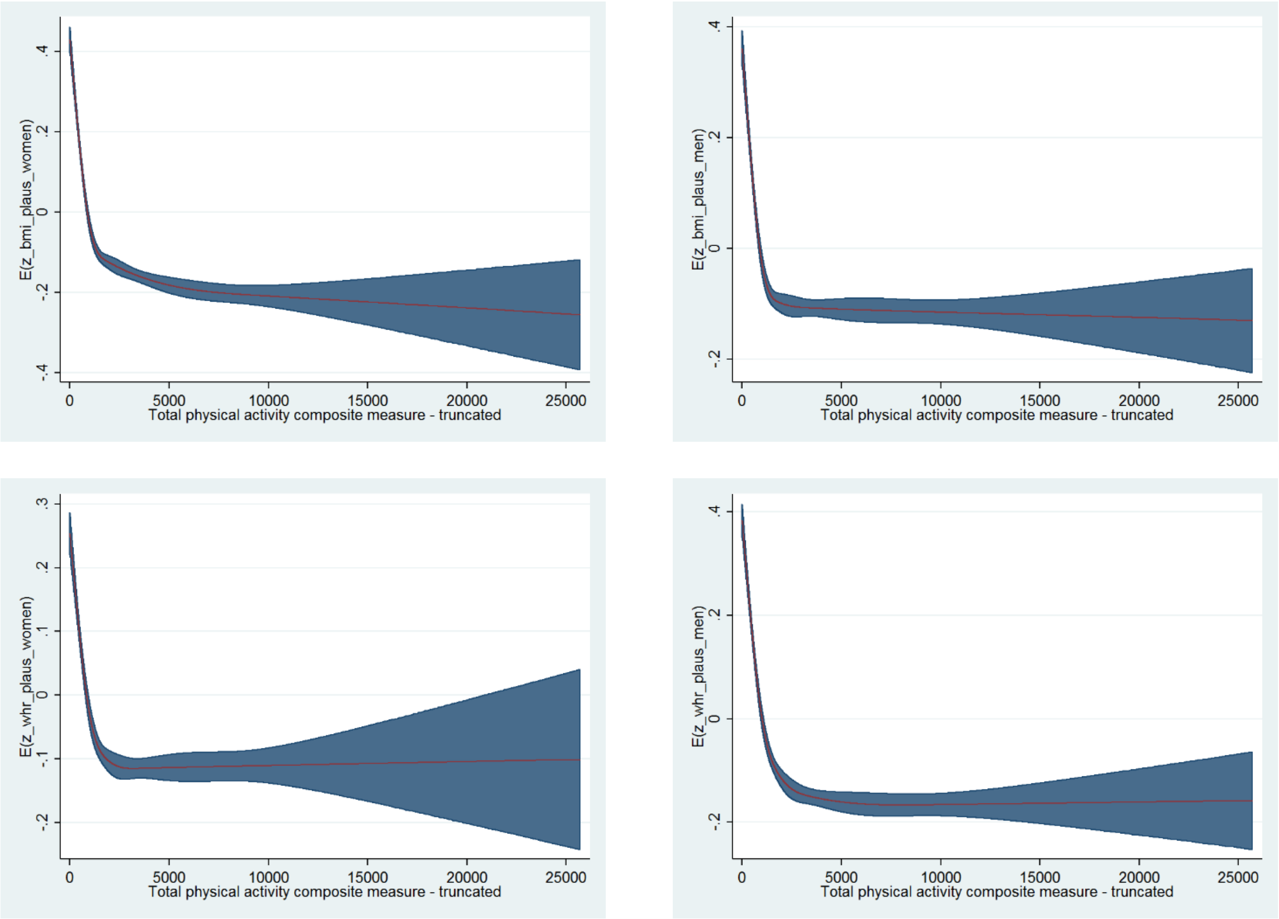

S1c Fig

Associations between social deprivation and adiposity measures allowing for cubic effects (regression curves allowing for restricted cubic splines): (a) BMI in women (top left); (b) BMI in men (top right); (c) WHR in women (bottom left); (d) WHR in men (bottom right).

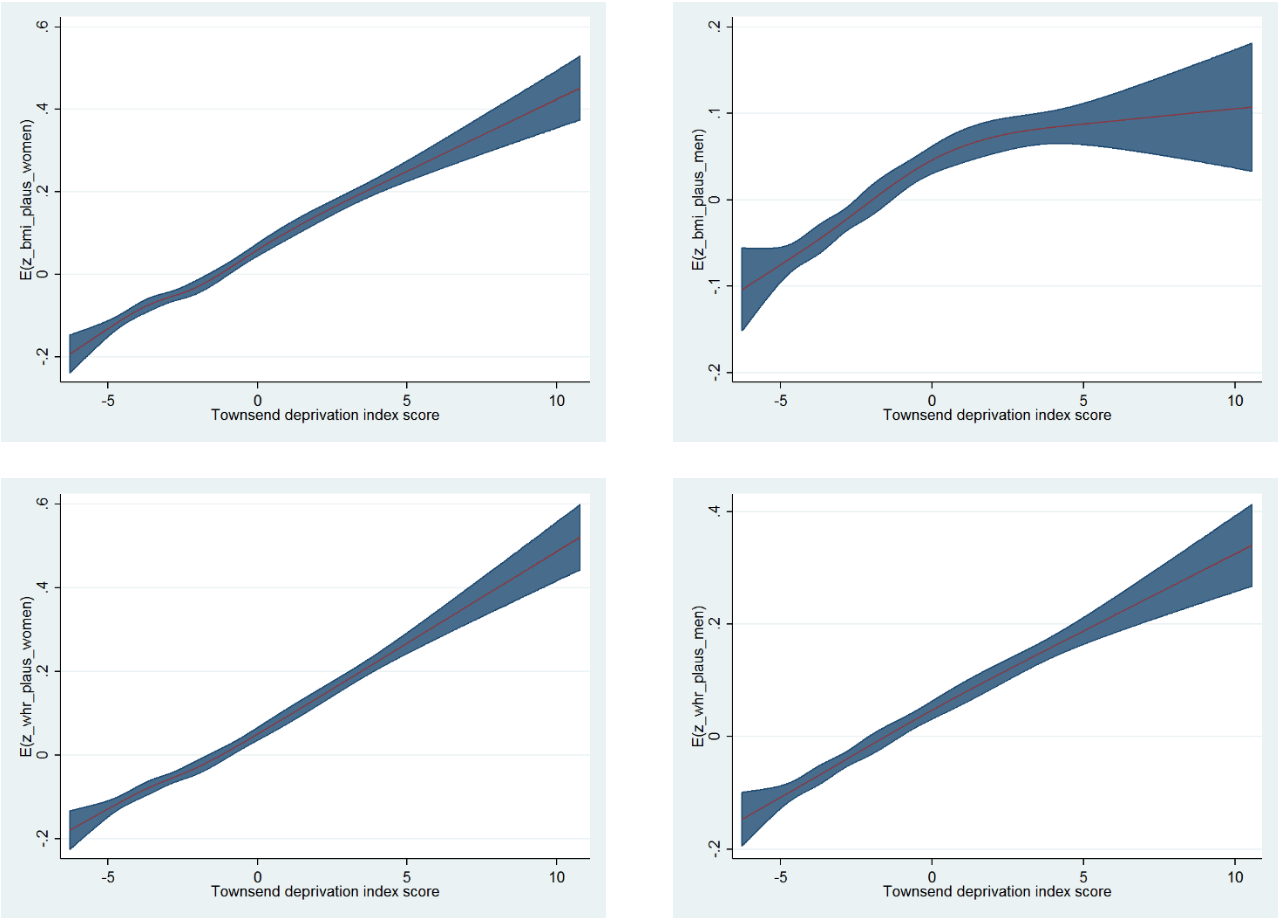

S1d Fig

Associations between cognitive reasoning ability and adiposity measures allowing for cubic effects (regression curves allowing for restricted cubic splines): (a) BMI in women (top left); (b) BMI in men (top right); (c) WHR in women (bottom left); (d) WHR in men (bottom right).

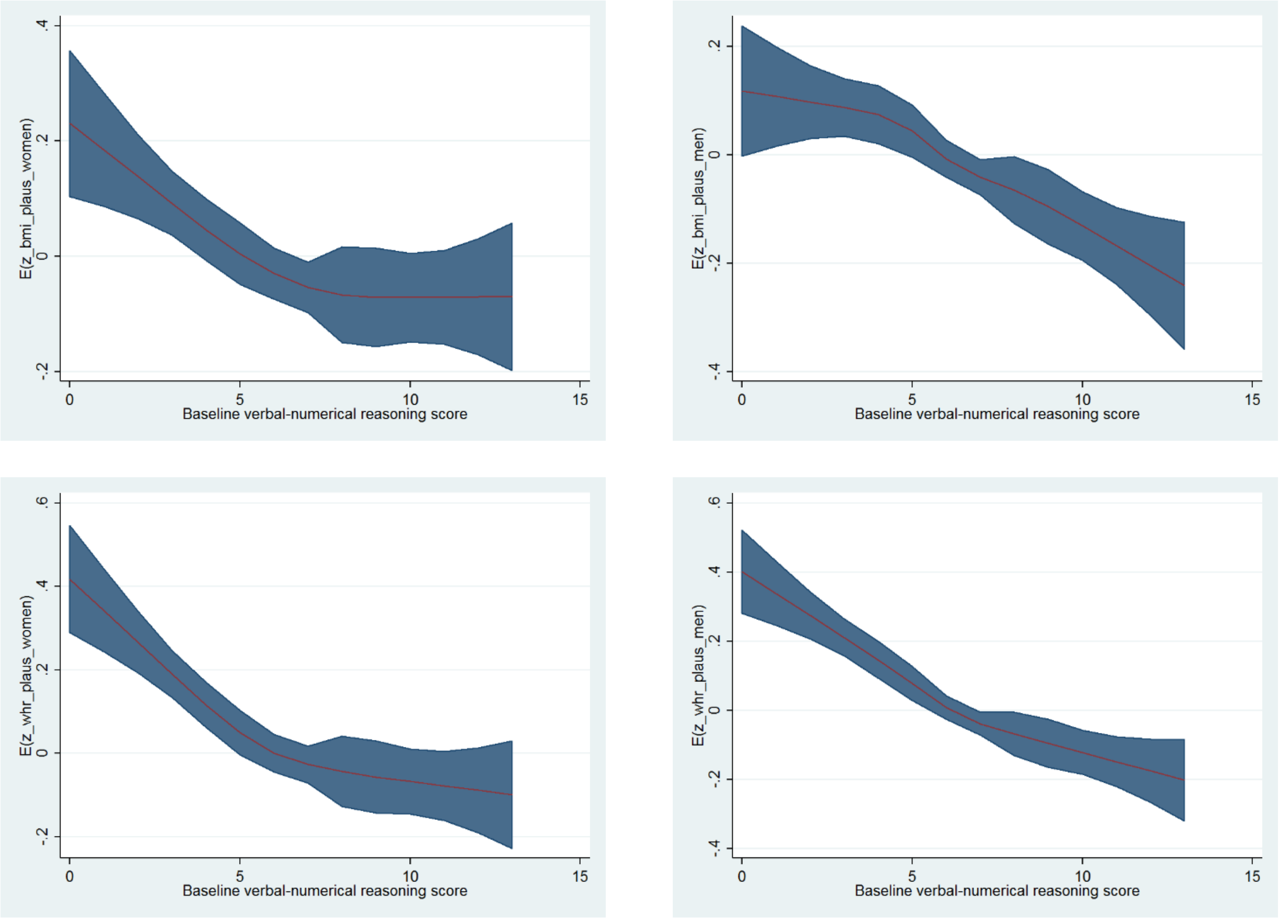

S1e Fig

Associations between neuroticism and adiposity measures allowing for cubic effects (regression curves allowing for restricted cubic splines): (a) BMI in women (top left); (b) BMI in men (top right); (c) WHR in women (bottom left); (d) WHR in men (bottom right).

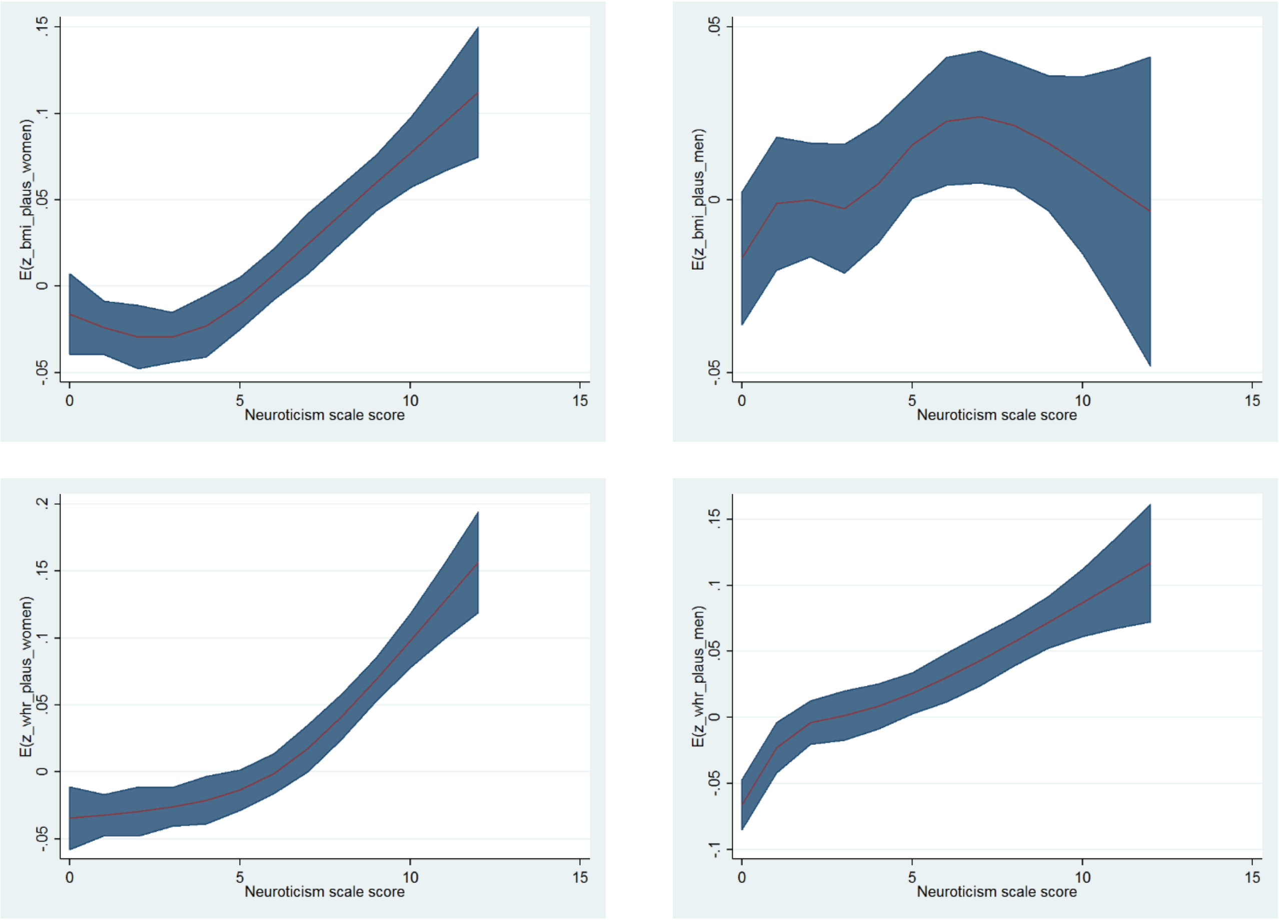

S2a Fig

Sex-specific associations between BMI PGS (SNP threshold p<.05) and adult BMI, by strata of lifestyle factors

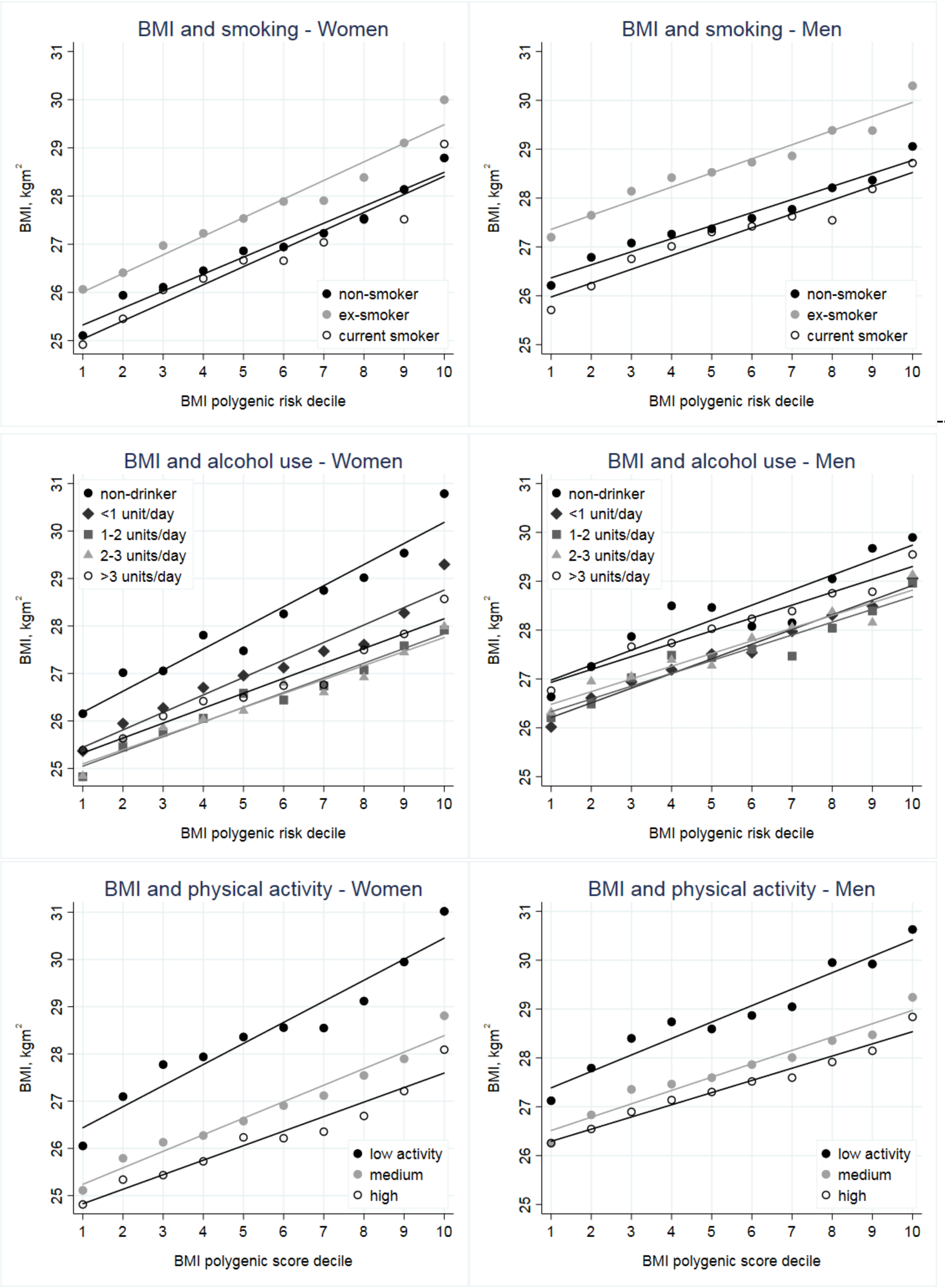

S2b Fig

Sex-specific associations between BMI PGS (SNP threshold p<.05) and adult BMI, by strata of socioeconomic status indicators

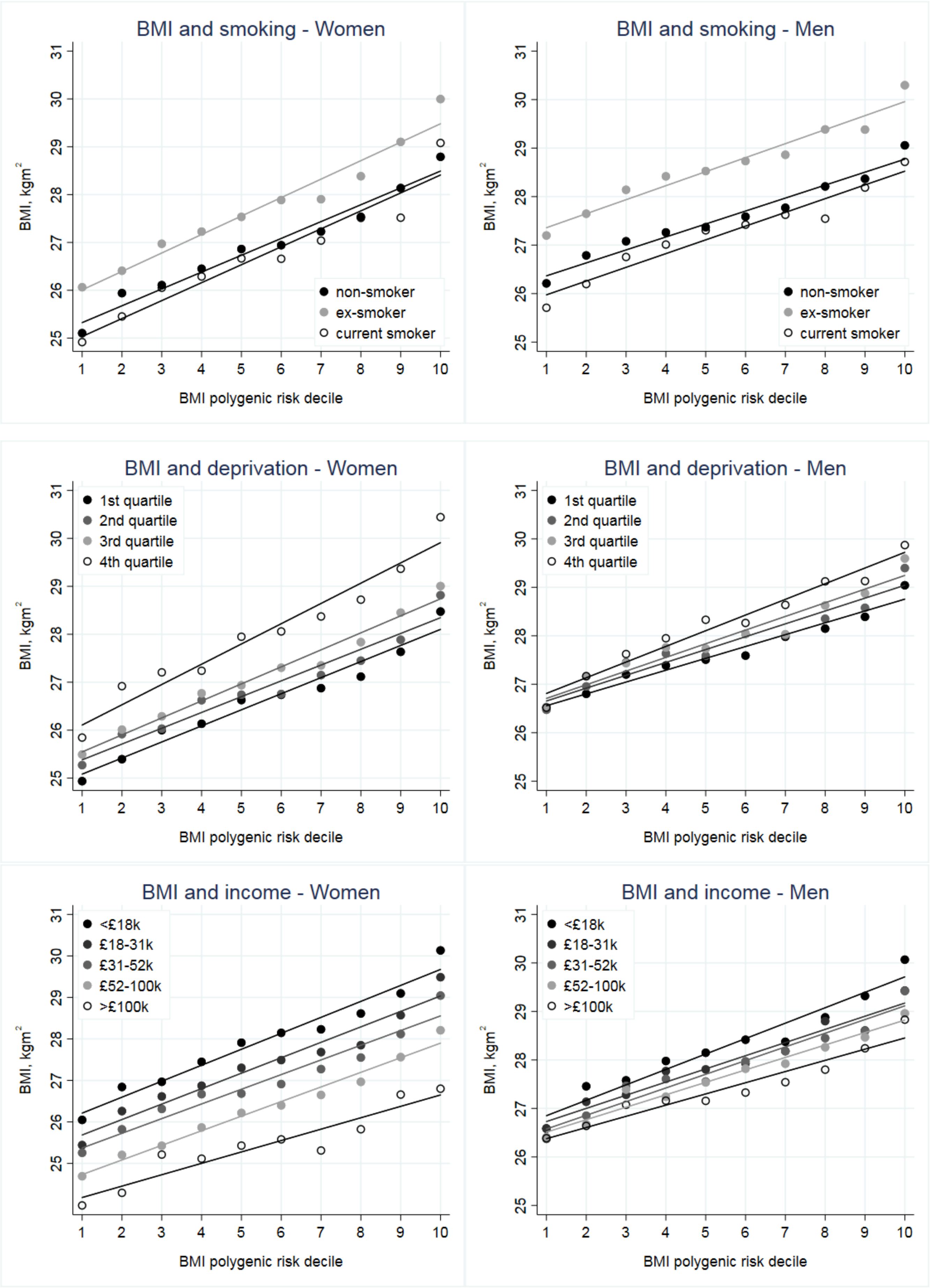

S2c Fig

Sex-specific associations between BMI PGS (SNP threshold p<.05) and adult BMI, by strata of psychological traits

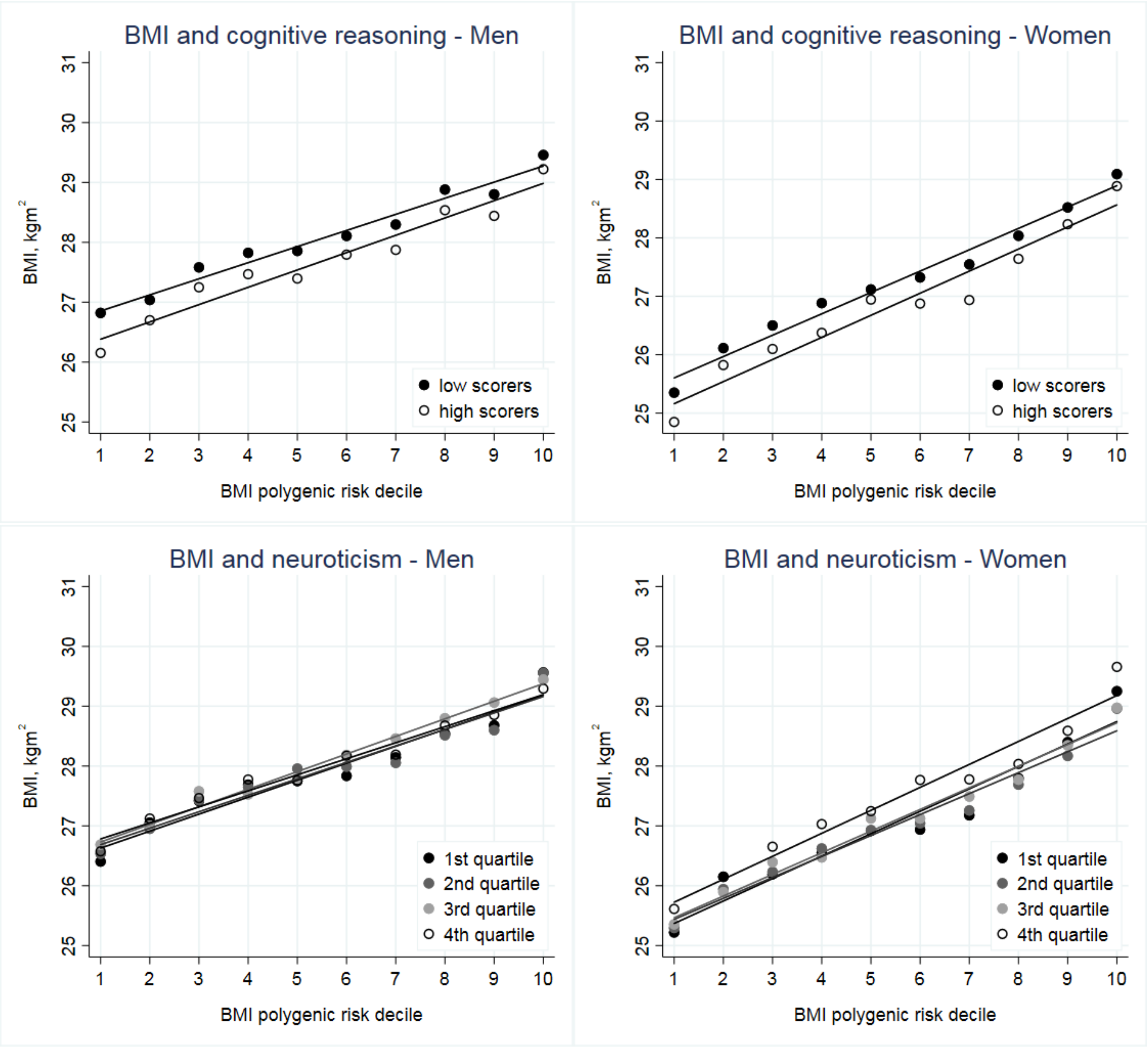

S3a Fig

Sex-specific associations between WHR_{adjBMI} PGS (SNP threshold p<.05) and adult WHR, by strata of lifestyle factors

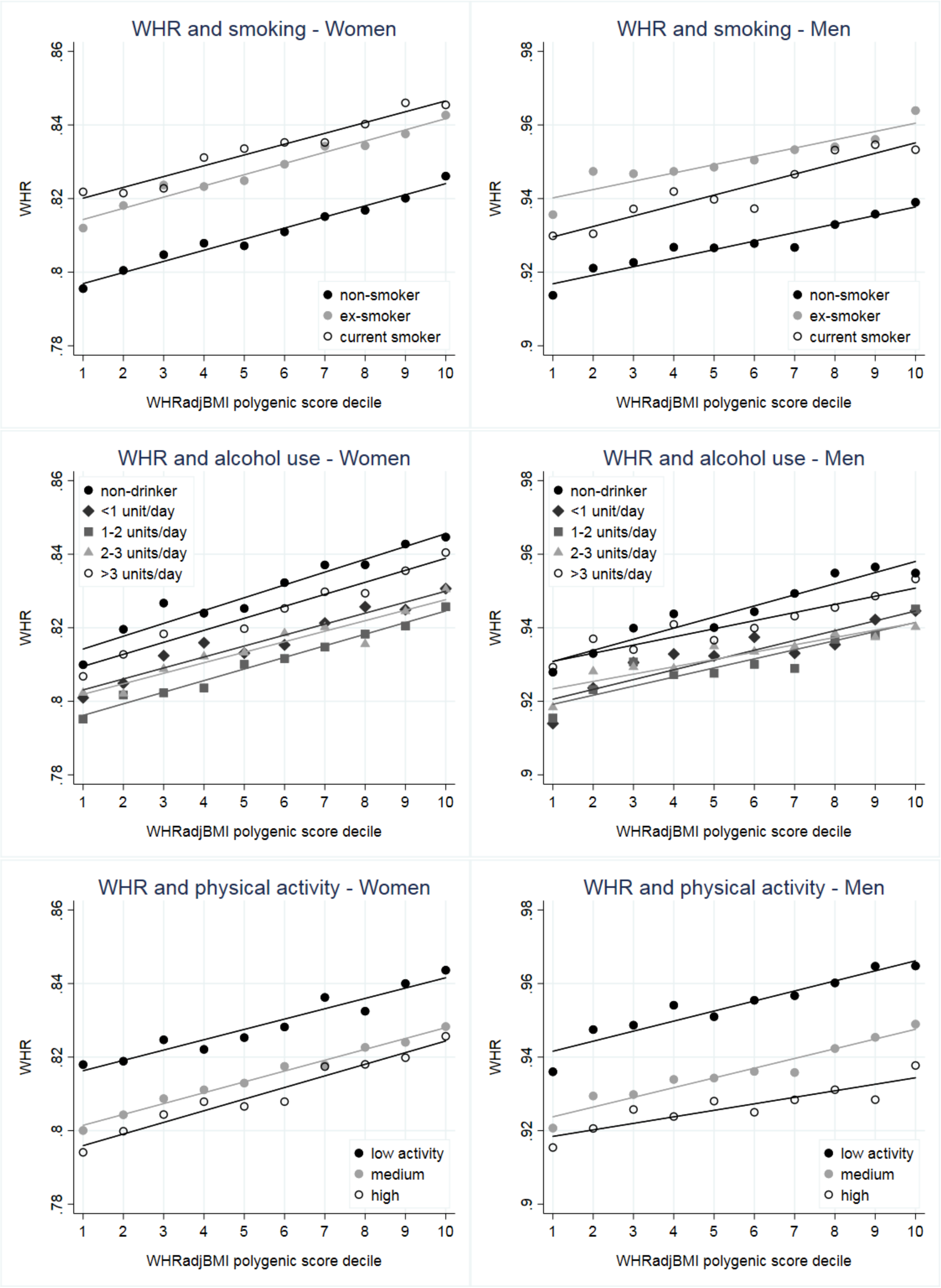

S3b Fig

Sex-specific associations between WHR_{adjBMI} PGS (SNP threshold $p<.05$) and adult WHR, by strata of socioeconomic status indicators

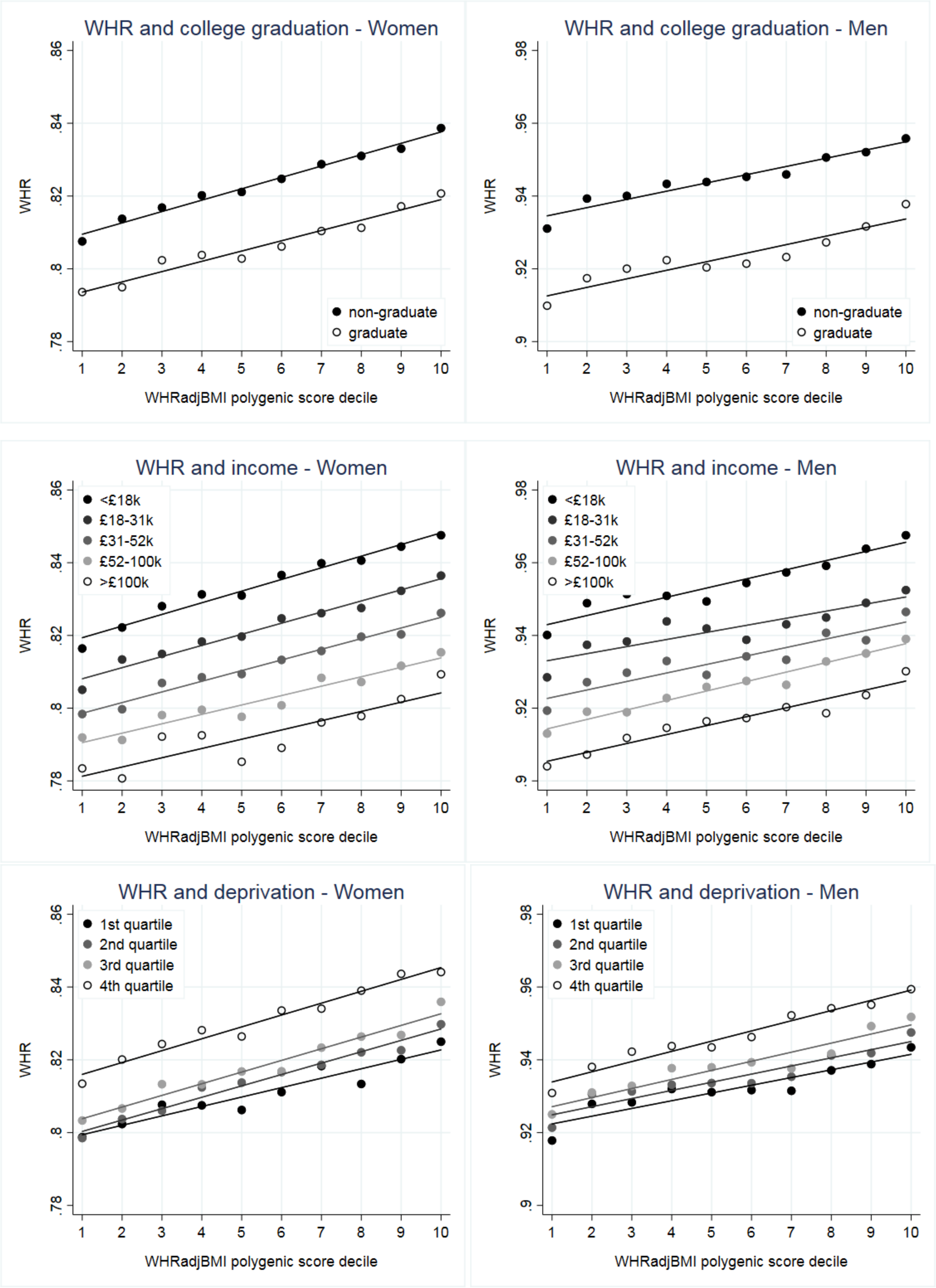

S3c Fig

Sex-specific associations between WHR_{adjBMI} PGS (SNP threshold $p<.05$) and adult WHR, by strata of psychological traits

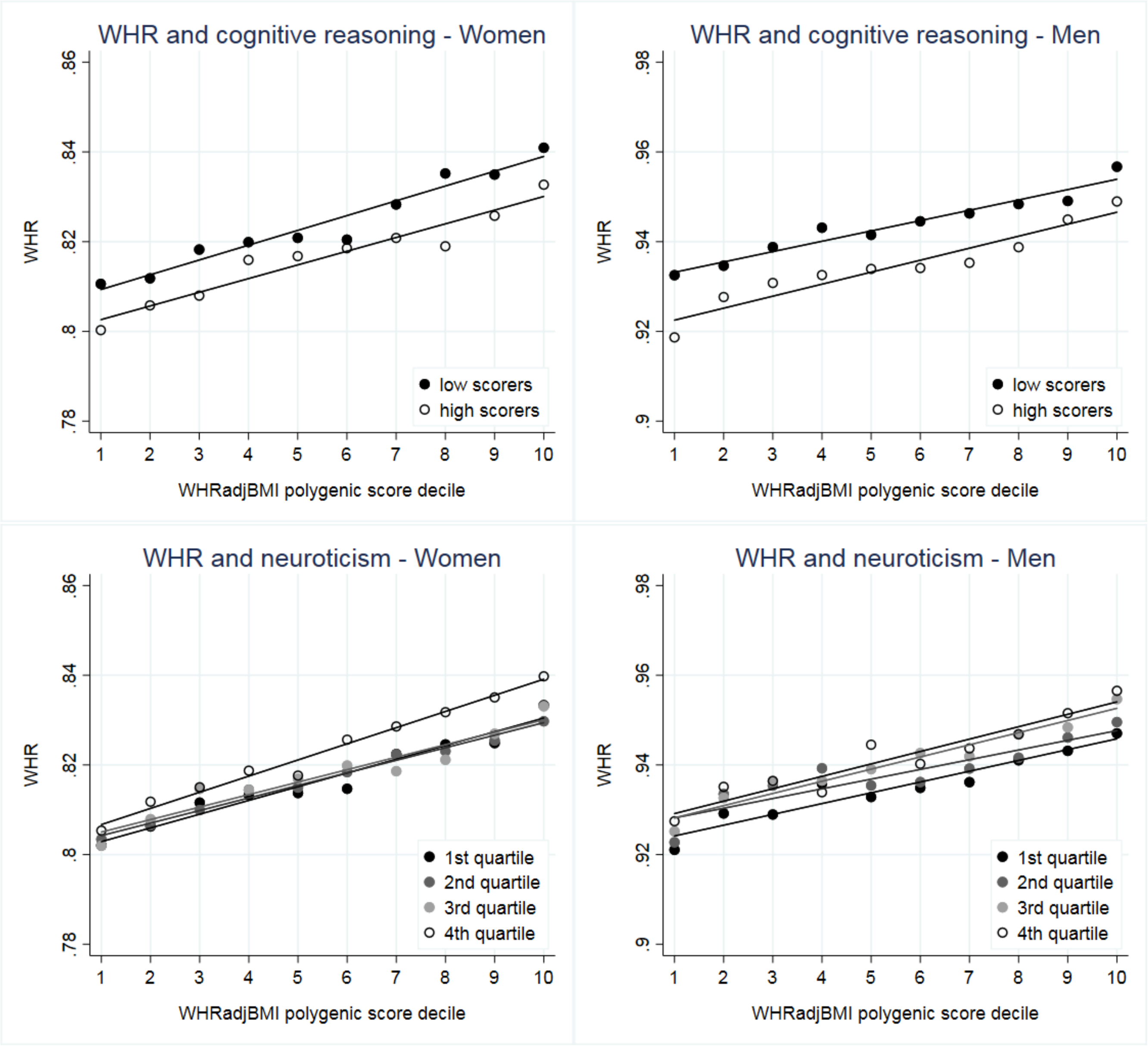

S4 Fig

Age and sex-specific associations between BMI PGS and adult BMI, by physical activity strata

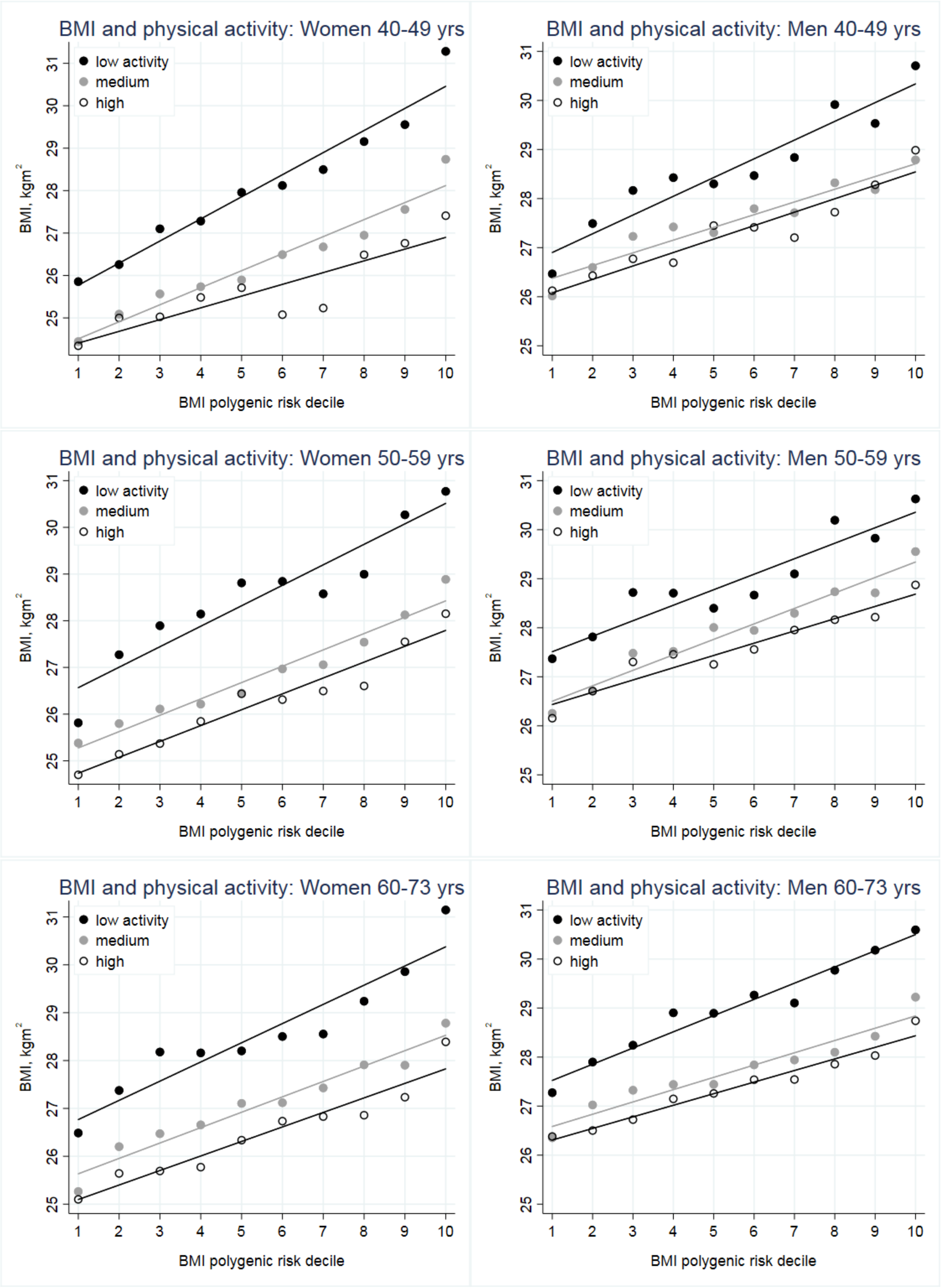

S5 Fig

Age and sex-specific associations between BMI PGS and adult BMI, by alcohol consumption strata

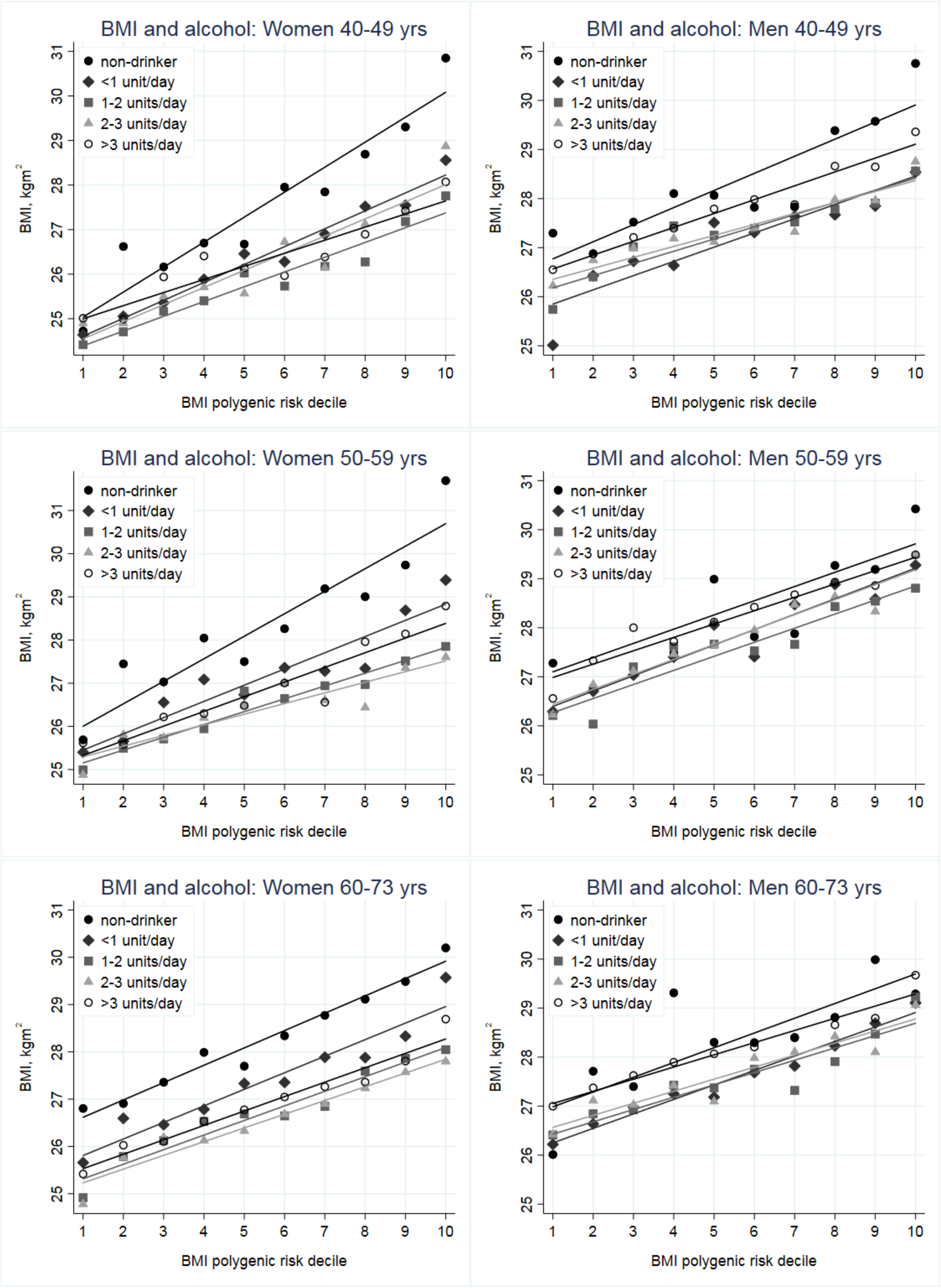

S6 Fig

Age and sex-specific associations between WHR_{adjBMI} PGS and adult WHR, by physical activity strata

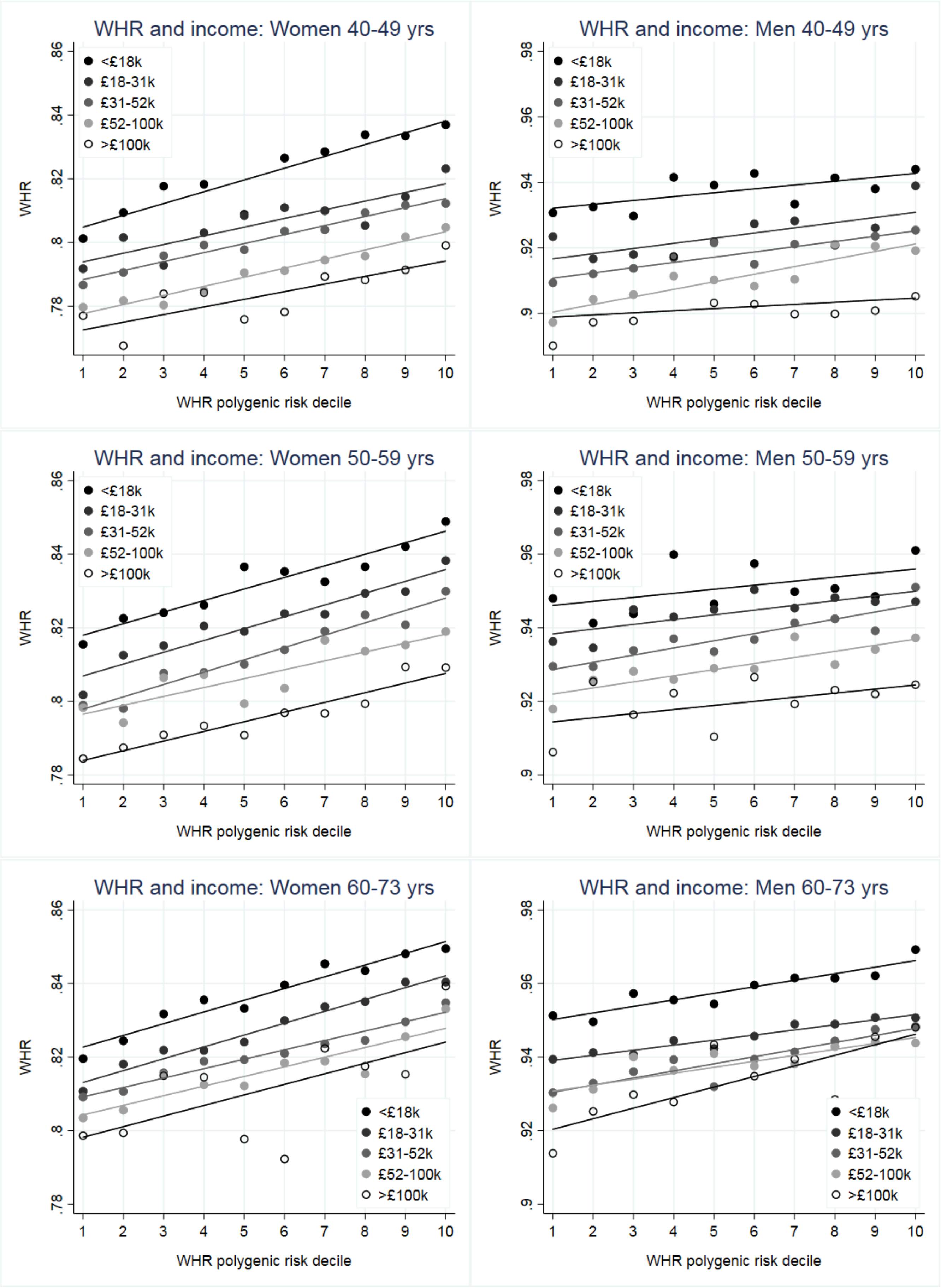

Supplement: Supplementary file 1 — Supplementary materials [file 41598_2018_36629_MOESM1_ESM.pdf]
